# NAD^+^ and Sirt5 restore mitochondrial bioenergetics failure and improve locomotor defects caused by *sucla2* mutations

**DOI:** 10.1172/jci.insight.181812

**Published:** 2026-01-23

**Authors:** Joy Richard, Giulia Lizzo, Noélie Rochat, Adrien Jouary, Pedro T.M. Silva, Alice Parisi, Stefan Christen, Sofia Moco, Michael B. Orger, Philipp Gut

**Affiliations:** 1 Nestlé Institute of Health Sciences, Nestlé Research, Switzerland.; 2 Champalimaud Research, Champalimaud Centre for the Unknown, Portugal.; 3 Institute of Food Safety and Analytics, Nestlé Research, Switzerland.

**Keywords:** Cell biology, Metabolism, Genetic diseases, Mitochondria

## Abstract

Mitochondria-derived acyl-coenzyme A (acyl-CoA) species chemically modify proteins, causing damage when acylation reactions are not adequately detoxified by enzymatic removal or protein turnover. Defects in genes encoding the mitochondrial respiratory complex and TCA cycle enzymes have been shown to increase acyl-CoA levels due to reduced enzymatic flux and result in proteome-wide hyperacylation. How pathologically elevated acyl-CoA levels contribute to bioenergetics failure in mitochondrial diseases is not well understood. Here, we demonstrate that bulk succinylation from succinyl-CoA excess consumes the enzymatic cofactor NAD^+^ and propagates mitochondrial respiratory defects in a zebrafish model of succinyl-CoA ligase deficiency, a childhood-onset encephalomyopathy. To explore this mechanism as a therapeutic target, we developed a workflow to monitor behavioral defects in *sucla2^–/–^* zebrafish and show that hypersuccinylation is associated with reduced locomotor behavior and impaired ability to execute food hunting patterns. Postembryonic NAD^+^ precursor supplementation restores NAD^+^ levels and improves locomotion and survival of *sucla2^–/–^* zebrafish. Mechanistically, nicotinamide and nicotinamide riboside require the NAD^+^-dependent desuccinylase Sirt5 to enhance oxidative metabolism and nitrogen elimination through the urea cycle. Collectively, NAD^+^ supplementation activates Sirt5 to protect against damage to mitochondria and locomotor circuits caused by protein succinylation.

## Abstract 2

Inherited defects in mitochondrial energy generation due to excessive production of reactive succinyl-CoA molecules can be counteracted by restoring NAD^+^ metabolism.

## Introduction

The coupling of neuronal sensory functions with directed locomotion is a fundamental biological process that enables complex behaviors of animals. Mutations in genes that cause defects in mitochondrial oxidative metabolism often manifest with pathologies in the brain and skeletal muscle, reflecting the high energy demand of these tissues to execute locomotor behavior (1–3). In human mitochondrial diseases, a group of inherited multisystem disorders that have a reduced electron transport chain function in common, the most frequent clinical presentation is encephalomyopathic symptoms (1, 3). However, the onset and the severity of these symptoms are highly variable. This phenotypic variability is difficult to explain through the inborn biochemical defect alone (1, 2, 4). Indirect pathological mechanisms that over time add on to the primary perturbation, for example, excessive production of ROS, have been proposed to contribute to the progression of mitochondrial diseases (5, 6).

Posttranslational modifications of proteins from mitochondria-derived acyl-coenzyme A (acyl-CoA) species are emerging as an important alternative source of cellular damage (7–12). Protein acylation is caused by locally enriched levels of acyl-CoA species through nonenzymatic chemical reactions, a process that is referred to as carbon stress (7–9). Acylation modifications can affect protein functions by interfering with catalytic sites of enzymes, protein-protein interactions, or protein stability (7–15). Proteins within mitochondria are particularly exposed to acylation modifications because the formation of covalent bonds with lysine residues is favored by the high levels of acyl-CoA metabolites and the basic pH within the organelle (9, 11, 15). In physiological conditions, most forms of acylation are proposed to occur at a low stoichiometry (15–18). An excessive buildup of protein acylation is prevented by sirtuin proteins, a group of NAD^+^-dependent deacylases that remove acyl marks from lysine residues (10, 15, 17, 18). Out of the 7 mammalian sirtuins, SIRT3, SIRT4, and SIRT5 locate in the mitochondria, where they have different substrate specificities (11, 12). In addition to the control of protein acylation by sirtuins, less specific cellular processes are likely important to prevent a harmful accumulation of acylation modifications. For example, proliferative tissues and cell lines have lower levels of protein acylation compared with nondividing cells, suggesting that protein turnover plays a role in reducing acylation load (15, 17). Taken together, when acylation events overcome cell-intrinsic capacities to remove acylation sites or to replace modified proteins, potentially irreversible damage can occur (10).

Compared with the well-established role of sirtuins to control cellular protein acylation, the impact of excessive acyl-CoA species from defective mitochondria as a source of damage is less well understood. We previously reported that protein succinylation is a widespread posttranslational modification that modifies hundreds of lysine residues across the proteome of cells derived from patients with succinyl-CoA ligase (SCL) deficiency (19). SCL deficiency is a mitochondrial disease caused by mutations in *SUCLA2*, which encodes the catalytic subunit expressed mainly in brain and muscle, or *SUCLG1,* the noncatalytic subunit forming the heterodimeric backbone of the enzyme complex (OMIM #612073 and #245400) (20, 21). Clinically, SCL deficiency manifests as methylmalonic aciduria and Leigh/Leigh-like encephalomyopathy, including hypotonia, dystonia, and deafness (20–23). Strikingly, we observed that protein succinylation directly and substantially contributes to impaired mitochondrial oxidative function in a zebrafish model of SCL deficiency.

The conservation of this mechanism in zebrafish makes it an attractive tool to identify new therapeutic strategies against damage from excessive acyl-CoA species on disease symptoms reported in patients, including impaired locomotor abilities (1, 3, 19, 22). Zebrafish have unique advantages for behavior profiling. Their small size makes them amenable to movement tracking in a multi-well format, allowing the rapid identification of genes or small molecules that modulate complex behavior traits in live vertebrates (24, 25). For example, tactile, acoustic, or visual stimuli evoke behavioral responses in zebrafish larvae that require coupling of neuronal sensory signals to motor output and that are, at least for the most basic phenotypes, relatively straightforward to measure (26–32).

In this study, we showed that *sucla2^–/–^* zebrafish develop the ability to respond to various stimuli with normal ranges of motion across different stereotypical movements necessary for swimming, albeit with a lower baseline activity and an impaired ability to perform food hunting. Since locomotor defects are common in mitochondrial diseases, we set out to exploit behavioral phenotypes in this vertebrate disease model of SUCLA2 deficiency for in vivo discovery of new therapeutic approaches. To this end, we developed a workflow to noninvasively select autosomal recessive carriers of *sucla2* gene defects for rapid downstream experimentation in hundreds of animals using a sensitive redox dye. As the main phenotypic readout, we chose a setup that quantifies impaired light-evoked locomotor activity, hereafter referred to as light-flash response (LFR). Leveraging the increased throughput of the assay, we found that genetic defects in *sucla2* cause hypersuccinylation and depletion of NAD^+^ levels, a phenomenon we also observed with constitutive activation of *sirt5,* suggesting that excess succinylation reactions trigger NAD^+^ consumption. Supplementation of *sucla2^–/–^* zebrafish with nicotinamide (NAM) and nicotinamide riboside (NR) normalized NAD^+^ levels but depended on *sirt5* to restore food intake, mitochondrial respiratory functions, and locomotor behavior while promoting urea production.

## Results

### The sucla2^–/–^ zebrafish show hallmarks of human disease including impaired locomotor activity.

**Figure 1:**
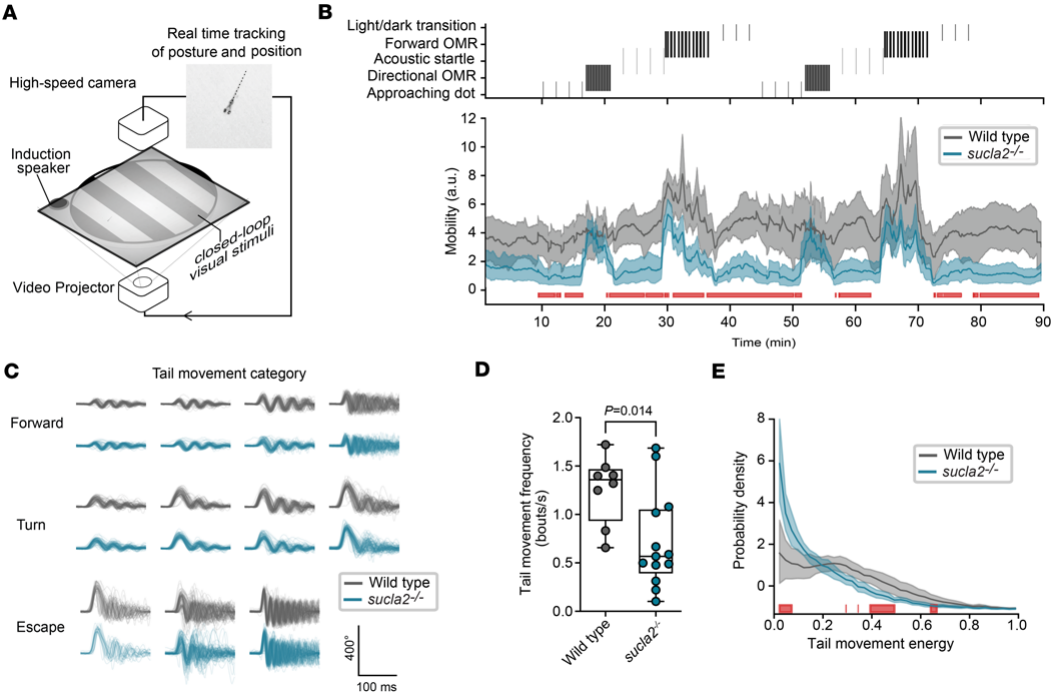
Behavior profiling of *sucla2^–/–^* larvae shows reduced locomotor activity. (**A**) Schematic representation of experimental setup for closed-loop presentation of visual and acoustic stimuli. (**B**) Sequence of visual and acoustic stimuli (top). Average mobility computed from the zebrafish trajectories in the open field arena (bottom). The shaded area represents the 99% confidence interval for the average estimated by bootstrapping. The red line indicates time periods when the confidence intervals of the WT and *sucla2^–/–^* zebrafish behavior did not overlap (WT *n* = 10, *sucla2^–/–^* = 17, 5–7 dpf). (**C**) Analysis of tail angle kinetics for 10 categories of movements (approach swim, slow 1, slow 2, burst swim, J turn, high angle turn, routine turn, spot avoidance turn, long latency C start, short latency C start; schematic overview of each movement category is in Supplemental Figure 2). For each category, 200 randomly selected movements are overlaid from the group of zebrafish per genotype. (**D**) Average frequency of tail bouts throughout the recording per genotype. The bar shows mean ± SEM. (**E**) Probability density function of the vigor of tail movements per genotype. The shaded areas indicate the 95% bootstrapped confidence interval. All analyses based on numbers per genotype as described for **B**.

Targeted gene disruption in the alpha subunit of the SCL complex, *sucla2,* causes a frameshift mutation and loss of protein expression (Supplemental Figure 1, A–C; supplemental material available online with this article; https://doi.org/10.1172/jci.insight.181812DS1). Like children affected by SCL disease, *sucla2^–/–^* zebrafish showed premature death, progressive loss of mtDNA content between 7 days after fertilization (dpf) and 10 dpf, increased lactate levels, and a reduced cellular ADP to ATP ratio concomitant with impaired oxidative metabolism (Supplemental Figure 1, D–K). Clinical symptoms of SCL deficiency are caused by impaired neuronal and muscle cell functions leading to encephalomyopathy. Locomotion is a behavior that depends on the coupling of signals from the CNS with contractions and directed movements of skeletal tissue. We applied an experimental paradigm that quantified locomotion in response to various stimuli as a surrogate readout for human locomotor defects, with the aim to test potential therapeutic effects of small-molecule or genetics-based interventions. To this end, we monitored swimming activity in an arena fitted with a high-speed camera as well as a projector and a speaker to expose the larvae to visual and auditory stimuli (Figure 1A). After 10 minutes of acclimatization during which spontaneous locomotion was monitored, consecutive light and acoustic stimuli were introduced, including transitions of light and dark cycles, moving grating to induce forward and directed swimming optomotor responses, acoustic-evoked startle behaviors, and a projected approaching dot simulating appearance of a predator that triggers an escape response (Figure 1B). These signals cause stereotypical locomotor movements that entail rapid episodes of propulsion known as tail bouts, followed by pauses, and that rely on different neuronal circuitries (Supplemental Figure 2A) (32).

While spontaneous swimming was reduced during acclimatization in *sucla2^–/–^* zebrafish, we observed robust tail bout responses across the set of stimuli (Figure 1B). To quantify the capability of *sucla2^–/–^* larvae to respond to stimuli, we employed a method that classifies tail bouts into 10 categories, providing a kinematic description of forward and turning movements and escape behaviors (Figure 1C and Supplemental Figure 2A) (32). Across these categories, *sucla2^–/–^* larvae were capable of activating tail bouts without differences in kinematics (Figure 1C). The intact locomotor kinetics suggest that *sucla2^–/–^* zebrafish do not develop substantial motor disorder during organogenesis. However, the frequency of bouts was substantially reduced together with a proportionally lower vigor of tail movements, together suggesting signs of reduced strength or movement volition (Figure 1, D and E). Reduced numbers of tail movements were particularly apparent in bout categories commonly used during routine swimming, including slow forward swimming and routine turns, further indicating a lower capacity to engage tail movements rather than a disability of the motor system per se (Supplemental Figure 2B). In summary, *sucla2^–/–^* zebrafish recapitulate hallmarks of human SCL deficiency including locomotor defects, making them a valid model for discovery of new therapeutic options.

### Noninvasive genotyping and light-evoked locomotion assay enable rapid phenotyping.

**Figure 2:**
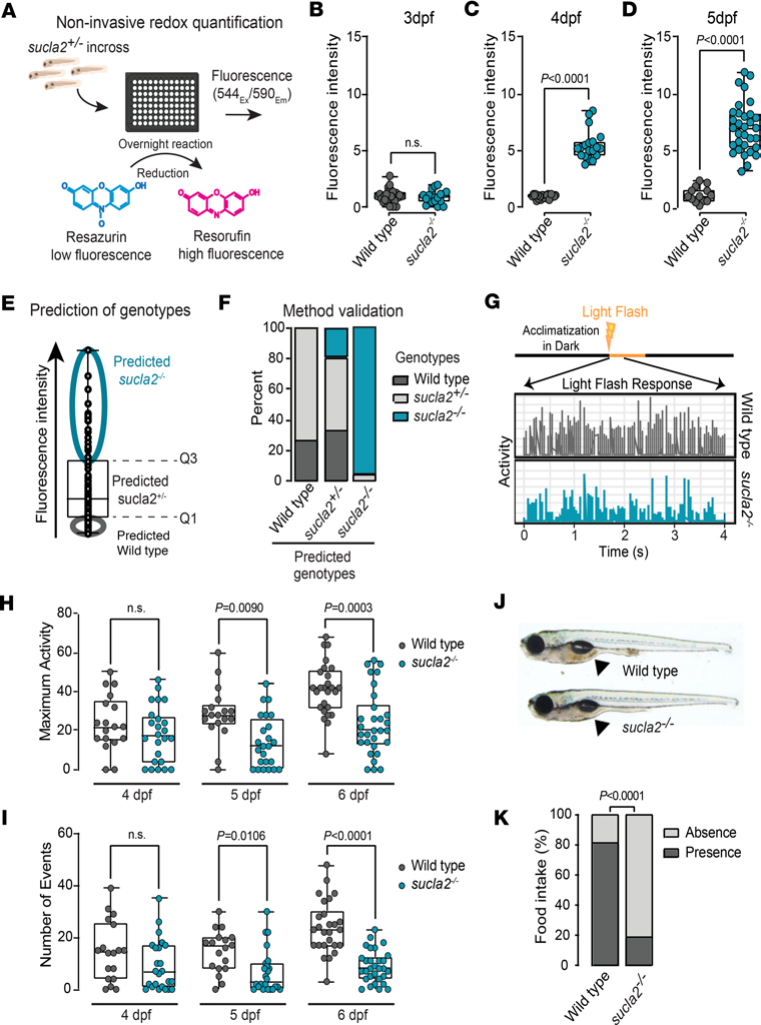
Noninvasive genotyping enables rapid profiling of light-evoked locomotion. (**A**) Illustration of noninvasive redox profiling: resazurin (blue) converted into pink, highly fluorescent resorufin based on cellular reduction capacity. Difference in reduction capacity between WT and *sucla2^–/–^* zebrafish was quantified in 96-well format. Ex, excitation; Em, emission spectra. (**B**–**D**) Fluorescence intensity in *sucla2^–/–^* and controls at (**B**) 3 dpf (WT, *n* = 28; *sucla2^–/–^*, *n* = 19), (**C**) 4 dpf (WT, *n* = 23; *sucla2^–/–^*, *n* = 19), and (**D**) 5 dpf (WT, *n* = 15; *sucla2^–/–^*, *n* = 32) after overnight treatment with resazurin. Results shown as fluorescence units normalized to WT values. *P* values calculated by 2-tailed unpaired parametric *t* test. (**E**) Genotype prediction for in-cross of *sucla2^+/−^* animals (*n* = 94, 5 dpf) after overnight resazurin treatment. Homozygous carriers of *sucla2* defects are predicted to be enriched in larvae pool with values above 75th percentile (fourth quartile). Q1, first quartile; Q3, third quartile. (**F**) Validation of discrimination of genotypes through HRM curve analysis. (**G**) Representation of protocol to LFR. Graph represents activity patterns of *sucla2^–/–^* and control zebrafish larvae during first 4 seconds of 10-second light exposure. (**H** and **I**) Quantification of maximal activity and number of movement events after light stimulus. Box plots show light-evoked movements during larval development: 4 dpf, 5 dpf (WT, *n* = 18; *sucla2^–/–^*, *n* = 24 for both time points), and 6 dpf (WT, *n* = 26; *sucla2^–/–^*, *n* = 30). *P* values (2-tailed) calculated by nonparametric Kruskal-Wallis test. (**J**) Images of *sucla2^–/–^* and control zebrafish larvae at 7 dpf. Black arrows highlight food in gastrointestinal tract. (**K**) Quantification of food intake in 7 dpf *sucla2^–/–^* and control zebrafish larvae (*n* = 26 per group). *P* value calculated by Fisher’s exact test. Box plots show median, Q1 to Q3, minima, maxima.

We next aimed to develop a scalable workflow for therapeutics discovery. To this end, we replaced the need for micro-biopsies from fin clips or euthanization of animals for genotyping by using a noninvasive method that can identify homozygous carriers of *sucla2* deletions in the absence of macroscopically obvious phenotypes for downstream experimentation. We hypothesized that noninvasive redox fluorescent dyes, commonly used to test cell viability, could be an attractive tool based on their sensitivity to assess changes in redox potential in inherited mitochondrial diseases (33). We added a low-fluorescent resazurin dye to larvae placed in 96-well microplates and determined the fluorescence emission of resorufin, the reduced form of resazurin, after an overnight incubation (Figure 2A). We found that *sucla2^–/–^* larvae generated a stronger fluorescence intensity compared with WT animals from 4 dpf onward (Figure 2, B–D). High-resolution melt (HRM) curve DNA analysis on the pools of zebrafish progeny from heterozygous adults confirmed that among larvae above the third quartile in fluorescence signal, nearly all animals were genotypically carrying *sucla2* deletions (Figure 2, E and F). The false-positive rate, defined as the presence of WT or heterozygous carriers of *sucla2* deletions in the top quartile, was below 5%, suggesting resorufin-based fluorescence generation as a robust noninvasive indicator of perturbed SCL activity (Figure 2, E and F, and Source Data File 1). Thus, this workflow enabled the selection of *sucla2^–/–^* zebrafish for rapid downstream experimentation.

Next, we developed a protocol for phenotyping locomotor defects using a simple light response assay to facilitate a higher experimental throughput. Instead of quantifying kinematics of tail bouts, we monitored locomotion spikes in a 96-well microplate reader with a camera. The stimulus was a light flash following a phase of dark acclimatization during which locomotion spikes were measured within the first 4 seconds, hereafter termed LFR. During this timeframe, an automated analysis script quantified the following metrics related to tail bout frequency and the vigor of movements similar to those observed with the behavioral arena: (a) the number of movement events and (b) the maximal amplitude of movement within 4 seconds per zebrafish (Figure 2G). Using this workflow, we performed time course experiments between 4 dpf and 6 dpf when zebrafish larvae transition from depletion of their yolk to a fasting state and start active food hunting (34, 35). The *sucla2^–/–^* zebrafish showed a reduced LFR in maximum activity and numbers of movement events compared with WT siblings at 5 dpf, a phenotype that was further pronounced at 6 dpf (Figure 2, H and I). These results suggest that light responses are perturbed in *sucla2^–/–^* larvae when WT animals begin active food seeking; that is, during hunting, zebrafish larvae visually detect food particles and activate a locomotor program to swim toward the food for ingestion (36). We tested whether *sucla2^–/–^* zebrafish showed limitations in finding food particles and found few food residues in the intestines of mutant carriers compared with controls (Figure 2, J and K). This impaired response to light stimuli is a quantifiable metric for locomotor defects that together with noninvasive genotyping can be used to test interventions.

### Reduced ability to capture prey in the absence of anatomical malformations.

**Figure 3:**
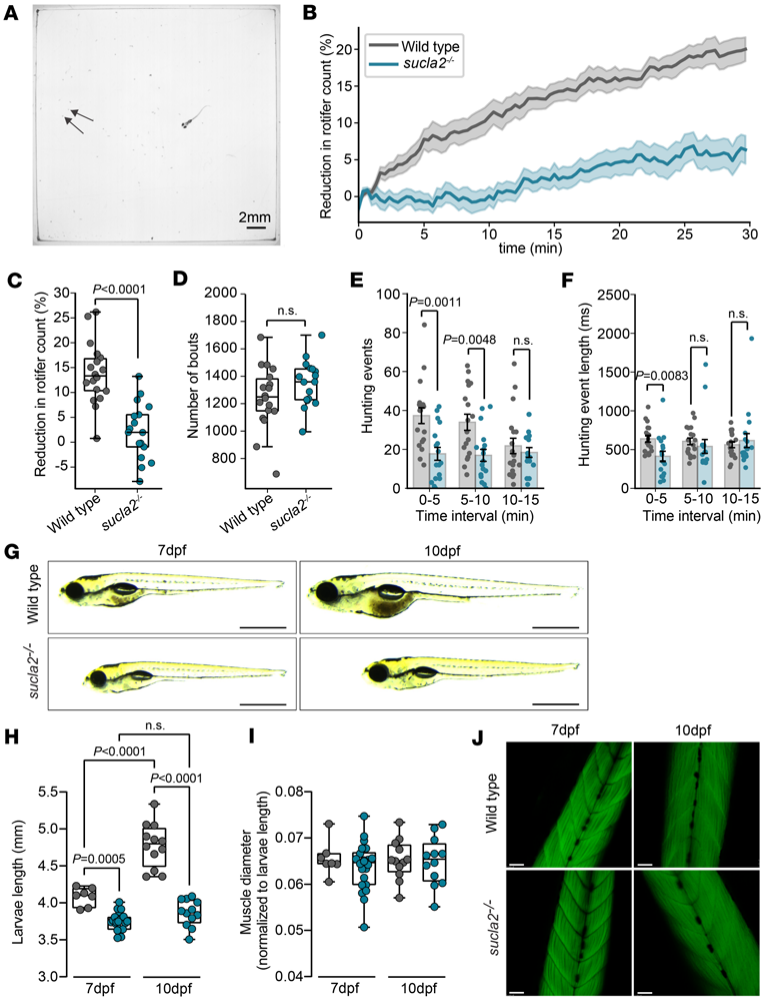
Prey capture ability is impaired in *sucla2^–/–^* larvae. (**A**) Top-down view of prey capture arena. Arrows indicate live rotifer prey. Scale bar: 2 mm. (**B**) Time series of rotifer count reduction in presence of 7 dpf *sucla2^–/–^* and control zebrafish larvae (WT, *n* = 18; *sucla2^–/–^*, *n* = 17), represented as mean ± SEM. (**C** and **D**) Quantification of reduction in rotifer count (**C**) and number of tail bouts (**D**) at 15-minute time point. (**E** and **F**) Number (**E**) and duration (**F**) of hunting events quantified over 5-minute intervals for the first 15 minutes of prey exposure. (**G**) Brightfield microscopy images of *sucla2^–/–^* and control zebrafish larvae at 7 and 10 dpf. (scale bars: 1 mm). (**H** and **I**) Biometrics quantification of (**H**) larvae length (mm) measured from mouth to tail and (**I**) muscle diameter measured from the end of gastrointestinal tract to upper part of body, and normalized to larvae length at 7 dpf (WT, *n* = 7; *sucla2^–/–^*, *n* = 22) and 10 dpf (WT, *n* = 12*; sucla2^–/–^*, *n* = 12). (**J**) Representative images of 7 dpf and 10 dpf larvae muscle fibers stained with phalloidin (scale bars: 50 μm). Statistics correspond to *P* value calculated by 2-tailed Mann-Whitney *U* test (**C**–**F**) or by ordinary 1-way ANOVA with Tukey’s multiple-comparison test (**H** and **I**). Box plots show median, first to third quartile, minima and maxima.

The inability to capture prey could be caused by issues in spotting prey or by anatomical malformations acquired during embryonic development that affect swimming. We turned to an open arena experiment in which free-swimming larvae were monitored alongside live rotifers, their natural prey (Figure 3A). The assay quantified hunting events when zebrafish larvae visually detect a rotifer as characterized by the convergence of eyes on the prey prior to a directional swim (32). In addition, high-resolution, high-speed cameras counted the reduction of rotifers in the arena as a readout for successful hunting events. Compared with WT zebrafish, *sucla2^–/–^* zebrafish consumed fewer rotifers throughout the experiment (Figure 3, B and C). No difference was detected between genotypes in the number of total tail bouts (Figure 3D). In contrast, *sucla2^–/–^* zebrafish initiated fewer hunting events with a moderately reduced length of such events during the first 5 minutes, followed by no differences between the groups at later time points (Figure 3, E and F). These results suggest that *sucla2^–/–^* larvae visually detect rotifers and process this information to initiate hunting patterns but do so at a reduced rate, leading to an overall decrease in food intake. This result excludes blindness as a cause and is consistent with observations that the *sucla2^–/–^* larvae utilize the full repertoire of tail movements but may lack stamina. This finding suggests underlying biochemical impairments in line with reduced ATP/ADP ratios, reduced mitochondrial respiratory rates, and elevated lactate (Supplemental Figure 1, G–K).

We further aimed to exclude anatomical malformation as a cause of impaired responses to neurobehavioral stimuli. Compared with WT animals, *sucla2^–/–^* larvae showed reduced growth rates (Figure 3, G and H). Despite the growth retardation, all anatomical structures appeared normal. Muscle diameter normalized to the length of the zebrafish did not differ between genotypes, suggesting a normal anatomical development of the tail muscle apparatus (Figure 3I). Phalloidin staining showed a normal somite structure and no differences in myofiber appearance between *sucla2^–/–^* larvae and their controls (Figure 3J).

Taken together, we found a reduced number of prey capture events, and the ability to identify rotifers and execute hunting programs was preserved in the absence of visible developmental defects.

### Pathological succinylation depletes NAD^+^ levels and impairs mitochondrial bioenergetics.

**Figure 4:**
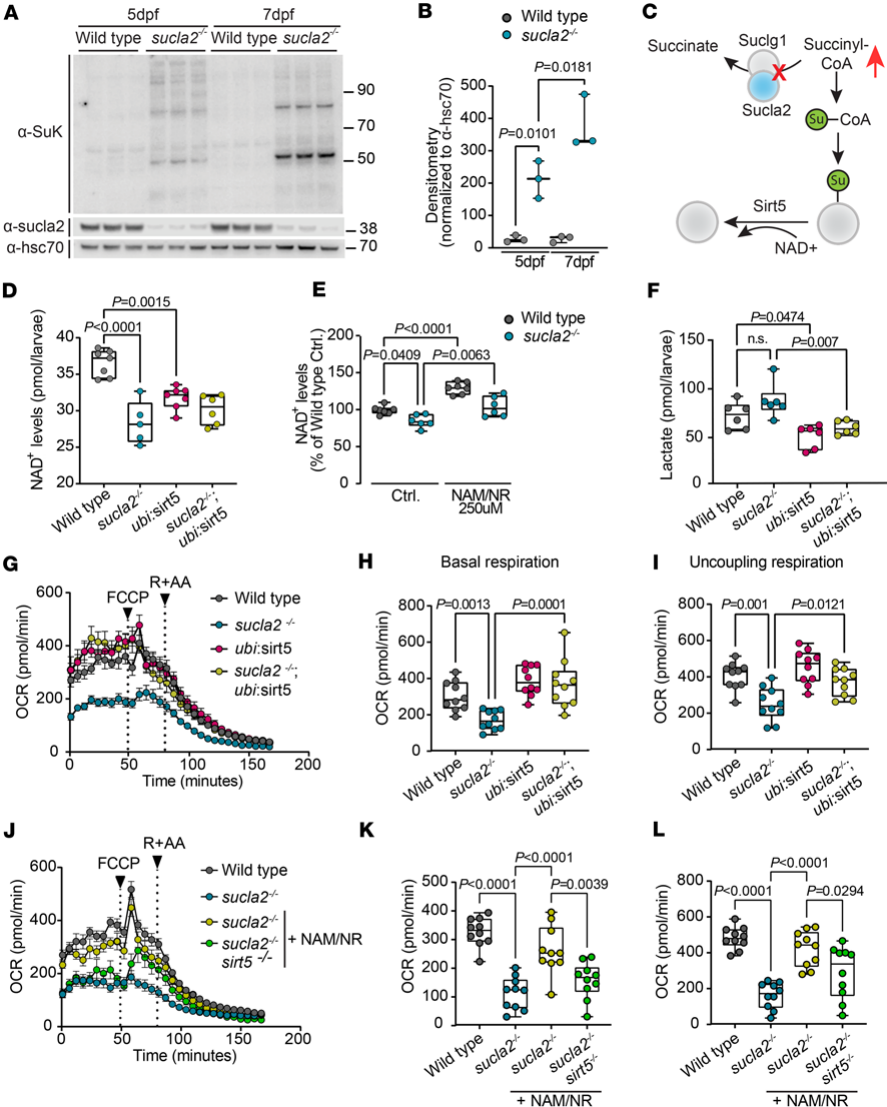
Mitochondrial respiratory failure is driven by succinylation-induced NAD^+^ depletion. (**A** and **B**) Global protein succinylation in *sucla2^–/–^* zebrafish larvae (*n* = 3 pools of 16 larvae) during larval transition from yolk-feeding to fasting. Box plots in **B** show quantifications of pan-succinyl-lysine Western blots using the mean of densitometry of 2 main bands normalized to Hsc70. (**C**) Mechanisms contributing to global protein succinylation in SCL deficiency. Succinylation occurs in response to reduced SCL flux and is removed by Sirt5, a NAD^+^-dependent desuccinylase. (**D**) NAD^+^ levels in WT and *sucla2^–/–^* animals with and without overexpression of *sirt5* (WT, *n* = 7; *sucla2^–/–^*, *n* = 5; *Tg*(*ubi:sirt5*), *n* = 7; *sucla2^–/–^ Tg*(*ubi:sirt5*), *n* = 6. Pool of minimum of 12 larvae, 7 dpf). (**E**) NAD^+^ levels in WT and *sucla2^–/–^* animals treated with 250 μM of NR and NAM for 40 hours. Data are relative to untreated WT control (WT, *n* = 7; *sucla2^–/–^*, *n* = 6; WT + NAM/NR, *n* = 7; *sucla2^–/–^* + NAM/NR, *n* = 6. Pool of minimum of 12 larvae, 7 dpf). (**F**) Lactate levels in *sucla2^–/–^* and controls with or without *sirt5* overexpression (*n* = 6 per genotype; pool of 11 larvae, 7 dpf). (**G**) Mitochondrial respiration measured by oxygen consumption rates (OCR) in *sucla2^–/–^* and controls with or without *sirt5* overexpression (*n* = 10 per group, 7 dpf) in fed conditions. FCCP is added to stimulate maximal OCR. Rotenone and antimycin A (R + AA) determine nonmitochondrial respiration. (**H**) Basal OCR calculated as the average of the 3 last points before addition of FCCP. (**I**) Maximal OCR in response to FCCP. (**J**) OCR levels in WT, *sucla2^–/–^*, and *sucla2^–/–^ sirt5^–/–^* zebrafish larvae treated with 250 μM of NAM/NR combination for 40 hours (*n* = 10 per group, 7 dpf) in fed conditions. (**K**) Basal OCR. (**L**) Maximal OCR. *P* values calculated by ordinary 1-way ANOVA with Tukey’s multiple-comparison test. Box plots show median, first to third quartile, minima and maxima.

Our findings suggest that the reduced locomotor activity of *sucla2*^–/–^ zebrafish may be caused by cellular energy shortage. Zebrafish activate molecular signatures of oxidative metabolism and pathways that rely on TCA cycle flux between 4 and 5 dpf (34, 35). These pathways include biosynthetic processes that require high cataplerotic metabolite flux (35, 37). We hypothesized that increased TCA flux would exacerbate succinylation because anaplerotic substrate flux will be throttled by genetic defects in SCL, thereby leading to excess succinyl-CoA. At 5 dpf, the time point when *sucla2^–/–^* larvae started showing impaired locomotor activity (Figure 2, H and I), protein succinylation was already detectable and significantly higher than in WT animals (*P* = 0.0101) (Figure 4, A and B). This signal increased further at 7 dpf in *sucla2^–/–^* zebrafish (*P* = 0.0181), whereas WT animals did not change in baseline succinylation levels (Figure 4, A and B).

In physiological conditions, low-grade nonenzymatic tagging of proteins by reactive succinyl-CoA is counteracted by the activity of the NAD^+^-dependent deacylase SIRT5 (17, 19). Excessive levels of succinyl-CoA may only become toxic to the cell once the capacity of SIRT5 to remove succinylation modifications is exhausted through a bulk succinylation effect or additional factors that reduce Sirt5 functions (Figure 4C). Based on the latter possibility, we hypothesized that succinylation reactions from abundantly available succinyl-CoA may lead to a chronic activation of Sirt5 and depletion of NAD^+^, a cofactor that is required for its activity (7, 12). Specifically, deacylation reactions lead to the cleavage of NAD^+^ into NAM, and the acyl-moiety forms 2’-acyl-ADP-ribose (38). The enzymatic activity of sirtuins is sensitive to a decline in intracellular NAD^+^ levels, suggesting that depletion of NAD^+^ may contribute to inadequate endogenous Sirt5 activity in SCL disease (38, 39). NAD^+^ levels of *sucla2^–/–^* zebrafish larvae were indeed substantially lower when compared with that of healthy controls (Figure 4D). Overexpression of *sirt5* in WT animals was sufficient to decrease NAD^+^ levels, consistent with consumption of NAD^+^ by Sirt5 (Figure 4D). The combined genetic background in *Tg(ubi:sirt5;cryaa:zsgreen) sucla2^–/–^* zebrafish did not further deplete NAD^+^ levels (Figure 4D). These data suggest that a decline of NAD^+^ levels limits endogenous Sirt5 functions, and that NAD^+^ levels constitute a potential therapeutic target in SCL deficiency.

NAD^+^ levels can be restored by precursors that use de novo biosynthetic and salvage metabolic pathways (40). To identify whether NAD^+^ levels can be restored through supplementation with vitamin B_3_ precursors, we screened candidate molecules in WT larvae to identify those with the highest potential to restore NAD^+^ status. All precursors tested at 500 μM, including nicotinamide mononucleotide (NMN), nicotinic acid (NA), NR, and NAM, significantly increased NAD^+^ levels (*P* < 0.0001 for all comparisons) (Supplemental Figure 3A). We selected NR and NAM as a combinatory treatment that increases NAD^+^ through 2 mechanisms: we incubated zebrafish with 250 μM of NR, a precursor that can be utilized by a pathway regulated by NR kinases (NRK1, NRK2), and 250 μM of NAM, a precursor of the NAD^+^ salvage pathway that is regulated by NAM phosphoribosyltransferase (40). SCL deficiency is a systemic disease that affects different organs. Precursors that increase NAD^+^ through different biosynthetic pathways may therefore be more effective to support NAD^+^ homeostasis across tissues accommodating their preferences in NAD^+^ generation (41). The combination of NAM/NR at 250 μM of each metabolite resulted in comparative levels of NAD^+^ compared with the single precursors at 500 μM (Supplemental Figure 3A). We next tested whether NAM/NR treatments at 250 μM each can efficiently increase NAD^+^ levels in *sucla2^–/–^* larvae and found a significant increase that restored the levels toward those in WT animals (*P* = 0.0063 sucla2*^–/–^* + NAM/NR vs. sucla2*^–/–^* vehicle treatments) (Figure 4E).

Next, we assessed whether restoring NAD^+^ level improves mitochondrial respiratory functions in SCL deficiency. We have previously shown that overexpression of *sirt5* in fasted *sucla2^–/–^* zebrafish larvae partially rescues mitochondrial respiratory functions (19). The inefficient rescue by genetic activation of *sirt5* may be due to a reduced availability of NAD^+^ as a cofactor. Alternatively, the rescue of mitochondrial respiration by Sirt5 may be limited by a lack of energy substrates from food. To this end, we tested whether provision of food could improve mitochondrial respiration capacity in *Tg(ubi:sirt5;cryaa:zsgreen) sucla2^–/–^* zebrafish larvae compared with *sucla2^–/–^* animals. Consistent with restored oxidative metabolism, overexpression of *sirt5* in the background of *sucla2^–/–^* deficiency lowered lactate levels compared with *sucla2^–/–^* larvae (Figure 4F). We found a striking rescue of baseline and uncoupling respiration, suggesting that energy substrates and precursors for NAD^+^ synthesis from food intake are necessary to restore mitochondrial respiratory functions in response to Sirt5 overactivation (Figure 4, G–I).

Next, we tested whether NAD^+^ supplementation can reactivate endogenous Sirt5 and reverse bioenergetics failure independent of genetic gain of function. Remarkably, treatment of *sucla2^–/–^* larvae with NAM/NR was sufficient to rescue mitochondrial respiration, suggesting that the depletion of NAD^+^ levels is limiting Sirt5 functions in SCL disease (Figure 4, J–L). Consistent with this hypothesis, the effects of NAM/NR were strongly blunted in *sirt5^–/–^ sucla2^–/–^* double KO (Figure 4, J–L). To test whether NAM/NR treatment changes bulk succinylation across the proteome, we analyzed pan-succinylation levels. Overall succinylation signals did not change, indicating that NAM/NR enables removal of a subset of succinyl-lysine modifications that are sufficient to restore critical cellular processes (Supplemental Figure 3, B and C).

Thus, our data identified a combination of exogenous NAM/NR as a strategy to restore mitochondrial bioenergetics in SCL deficiency through reactivation of endogenous Sirt5 enzymatic functions.

### Sirt5 and NAD^+^ activate amino acid metabolism and promote urea production.

**Figure 5:**
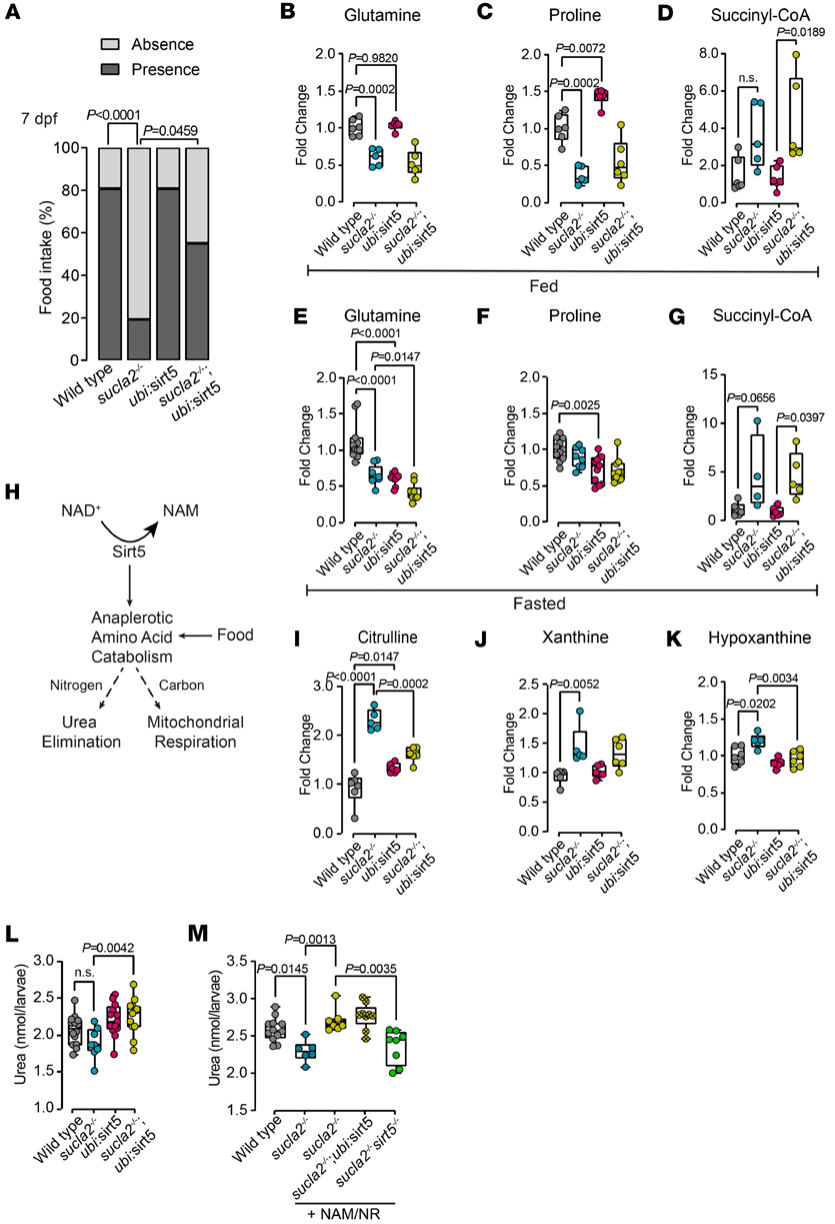
Sirt5 and NAD^+^ restore food intake and activate amino acid metabolism and urea generation. (**A**) Quantification of food intake in 7 dpf *sucla2^–/–^* and control zebrafish larvae with or without *sirt5* overexpression (WT; *sucla2^–/–^* *Tg(ubi:sirt5*), *n* = 26 per group; *sucla2^–/–^ Tg*(*ubi:sirt5*), *n* = 20). (**B**–**G**) Box plots showing fold changes of 2 anaplerotic amino acids: (**B** and **E**) glutamine and (**C** and **F**) proline; selected metabolites related to TCA cycle: (**D** and **G**) succinyl-CoA. (**H**) Simplified schematic representation of Sirt5-mediated amino acid catabolism. (**I**–**K**) Box plots showing fold changes of levels of selected metabolites related to nitrogen elimination: (**I**) citrulline, (**J**) xanthine, and (**K**) hypoxanthine. Experiments shown in **B**–**D** and **I**–**K** were performed under fed conditions (WT, *n* = 6; *sucla2^–/–^*, *n* = 5; *Tg*(*ubi:sirt5*), *n* = 6; *sucla2^–/–^ Tg*(*ubi:sirt5*), *n* = 6. Pool of 10 larvae, 7 dpf). Experiments shown in **E**–**G** were performed under fasted conditions. (**E** and **F**) WT, *n* = 11; *sucla2^–/–^*, *n* = 9; *Tg*(*ubi:sirt5*), *n* = 11; *sucla2^–/–^Tg*(*ubi:sirt5*); (**G**) WT, *n* = 6; *sucla2^–/–^*, *n* = 4; *Tg*(*ubi:sirt5*), *n* = 6; *sucla2^–/–^Tg*(*ubi:sirt5*), *n* = 5. Pool of 10 larvae, 7 dpf. (**L**) Urea levels from WT and *sucla2^–/–^* animals with and without overexpression of *sirt5* in fed conditions (WT, *n* = 14; *sucla2^–/–^*, *n* = 8; *Tg*(*ubi:sirt5*), *n* = 13; *sucla2^–/–^ Tg*(*ubi:sirt5)*, *n* = 12. Pool of minimum 12 larvae, 7 dpf). (**M**) Urea levels from WT and *sucla2^–/–^* zebrafish larvae with and without overexpression or KO of *sirt5*, immersed in egg water or treated with 250 uM of NAM/NR combination for 40 hours with access to food (WT, *n* = 12; *sucla2^–/–^*, *n* = 6; *sucla2^–/–^* + NAM/NR*, n* = 7; *sucla2^–/–^ Tg*(*ubi:sirt5*) + NAM/NR, *n* = 9; *sucla2^–/–^ sirt5^−/−^* + NAM/NR*,* *n* = 8. Pool of minimum 12 larvae, 7 dpf). Data in **E**, **F**, **l**, and **M** pooled from 2 independent experiments. Ordinary 1-way ANOVA with Tukey’s multiple- comparison tests used for all statistical testing. Box plots show median, first to third quartile, minima and maxima.

We next tested whether the restored bioenergetics capacity of *sucla2^–/–^* larvae in response to *sirt5* gain of function improved locomotor programs required for food intake. Overexpression of *sirt5* did not change food intake in WT controls but enabled food ingestion in *sucla2^–/–^* animals at 7 and 10 dpf (Figure 5A and Supplemental Figure 4A). Sirt5 has previously been described to regulate cellular metabolic processes (17). We therefore performed metabolomics experiments in fasted and fed WT and *sucla2^–/–^* zebrafish larvae to understand how Sirt5 activity improves cellular metabolism in the presence or absence of metabolic substrates. Strikingly, *sucla2^–/–^* carriers showed low levels of several amino acids, including the anaplerotic amino acids glutamine and proline compared with WT animals (glutamine, *P* = 0.0002; proline, *P* = 0.0002; WT vs. *sucla2^–/–^*), an effect that was not reversed when overexpressing *sirt5* in *sucla2^–/–^* larvae in the presence of food (Figure 5, B and C, and Supplemental Figure 4B). Sirt5 activation did not change levels of the 2 upstream metabolites of SCL, α-ketoglutarate (Supplemental Figure 4C) and succinyl-CoA (Figure 5D), indicating that Sirt5 gain of function does not overcome the primary enzymatic defect of the SCL complex (Figure 5D). In fasted conditions, glutamine was robustly depleted while proline did not differ between WT animals (Figure 5, E and F, and Supplemental Figure 4D), and α-ketoglutarate and succinyl-CoA remained unchanged (Figure 5G and Supplemental Figure 4E). Surprisingly, *sirt5* overactivation alone in the background of fasted WT larvae had a profound effect on amino acid metabolism: Sirt5 activation lowered the levels of a large set of amino acids, including that of glutamine and proline when compared with WT controls (Figure 5, E and F, and Supplemental Figure 4D). The combined genetic background of *Tg(ubi:sirt5;cryaa:zsgreen) sucla2^–/–^* animals further accentuated glutamine depletion and that of other amino acids compared with *sucla2^–/–^* carriers (Figure 5E and Supplemental Figure 5E). Together, these results suggest that *sucla2^–/–^* animals compensate for reduced SCL flux with depletion of amino acids, in particular glutamine, while Sirt5 activates catabolism of amino acids for mitochondrial respiration.

Mechanistically, if Sirt5 activation stimulates amino acid anaplerosis to support oxidative metabolism of the carbon backbone of amino acids, the nitrogen from the amino group needs to be eliminated to not cause accumulation of toxic ammonium (Figure 5H). Sirt5 has been shown to be required for urea cycle function in mammals, although the type of lysine modification removed by SIRT5 to reactivate urea flux may be different depending on the metabolic context (42, 43). Based on our findings, we hypothesized that Sirt5 is required to eliminate nitrogen from amino acid catabolism in SUCLA2 deficiency (Figure 5H). To this end, we analyzed metabolomics data from WT, *sucla2^–/–^*, *Tg(ubi:sirt5;cryaa:ZsGreen),* and *Tg(ubi:sirt5;cryaa:ZsGreen) sucla2^–/–^* animals in the presence of a protein-rich food source. Consistent with the hypothesis that Sirt5 facilitates urea cycle flux, we found that the levels of citrulline, which is the amino acid that binds an amino group from carbamoyl-phosphate (Supplemental Figure 4F), was significantly elevated in *sucla2^–/–^* animals when compared with WT animals (*P* < 0.0001) (Figure 5I). In contrast, *sirt5* gain of function efficiently lowered citrulline levels toward that of WT controls (Figure 5I). In addition, *sirt5* gain of function normalized levels of xanthine and hypoxanthine, suggesting a broad role in nitrogen elimination across metabolic pathways (Figure 5, J and K). To directly quantify the effects of Sirt5 on urea generation, we measured urea levels in whole larvae. As expected, *sirt5* overexpression led to a marked increase in urea generation in *Tg(ubi:sirt5;cryaa:ZsGreen) sucla2^–/–^* compared with *sucla2^–/–^* larvae (Figure 5L). NAM/NR supplementation replicated the genetic overexpression of *sirt5* by increasing urea excretion in *sucla2^–/–^* compared with controls (*P* = 0.0013) (Figure 5M). Like the Sirt5-dependent rescue of mitochondrial respiration, we found that lack of *sirt5* in the genetic background of *sucla2^–/–^* defects blunted the effects of NAM/NR, suggesting dependency on Sirt5 activity (Figure 5M).

In summary, these data demonstrate that Sirt5 and NAD^+^ supplementation promote amino acid utilization while enabling an efficient elimination of excess nitrogen.

### NAD^+^ precursors improve survival and restore light-evoked locomotion.

**Figure 6:**
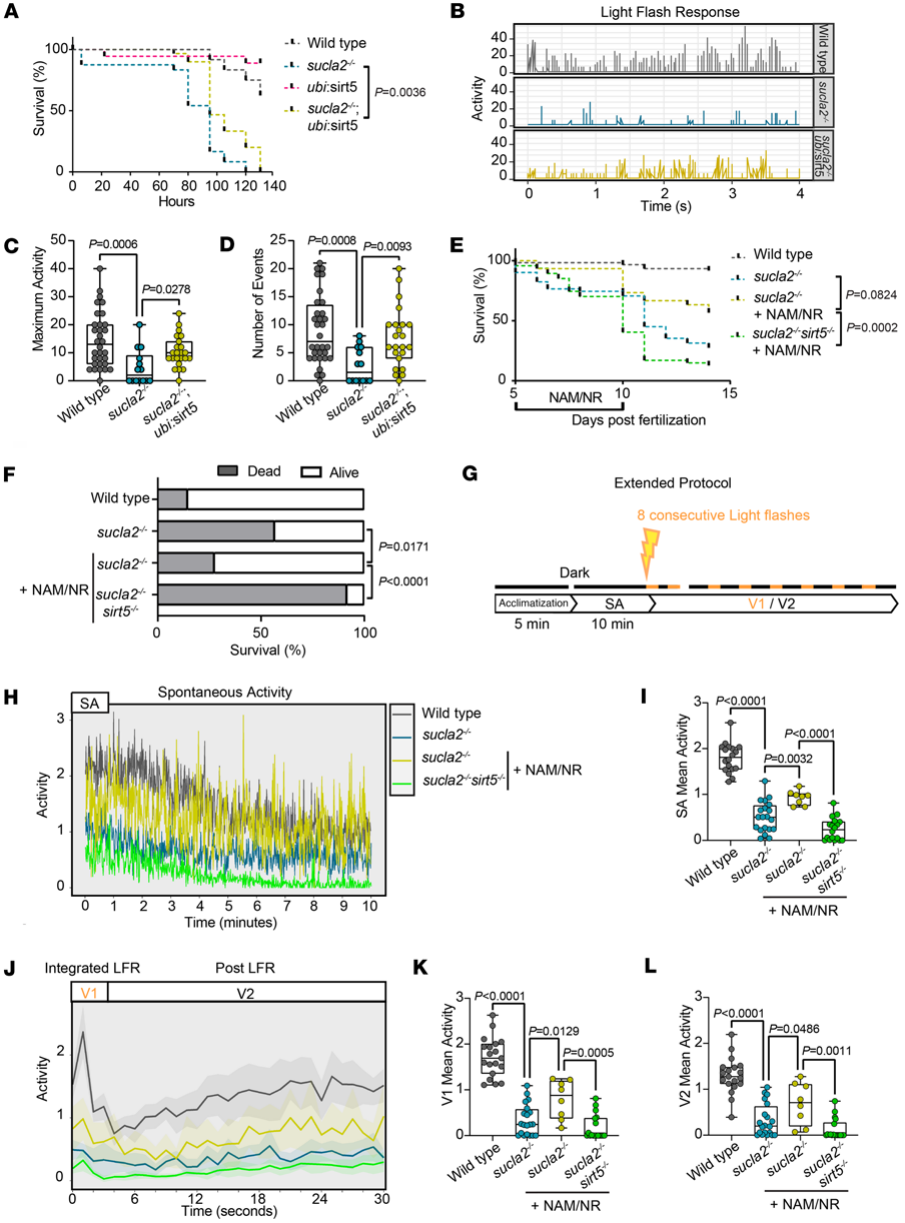
Sirt5 and NAD^+^ improve survival, spontaneous swimming, and LFR. (**A**) Survival and (**B**) LFR of *sucla2^–/–^* and control zebrafish larvae with or without *sirt5* overexpression (survival: WT, *n* = 24; *sucla2^–/–^*, *n* = 24; *Tg(ubi:sirt5*), *n* = 18; *sucla2^–/–^ Tg*(*ubi:sirt5*)*;* LFR: WT, *n* = 32; *sucla2^–/–^*, *n* = 14; *sucla2^–/–^ Tg*(*ubi:sirt5*), *n* = 23, 7 dpf, fed ad libitum 5–7 dpf). (**C** and **D**) Quantification of (**C**) movement amplitude per individual and (**D**) number of events in response to LFR. Data pooled from 2 independent experiments. *P* values calculated by nonparametric tests (Kruskal-Wallis, 2 comparisons). (**E**) Survival curve and (**F**) histogram of 14 dpf of WT and *sucla2^–/–^* zebrafish larvae with and without *sirt5^–/–^* genetic background, treated with NAM/NR from 5 to 10 dpf (WT, *n* = 60; *sucla2^–/–^*, *n* = 51; *sucla2^–/–^* + 250 μM NAM/250 μM NR*, n* = 30; *sucla2^–/–^ sirt5^–/–^* + 250 μM NAM/250 μM NR*,* *n* = 47, ad libitum fed). (**G**) Behavioral protocol: locomotor activity tracked for 10 minutes in dark condition. Spontaneous activity (SA) was quantified followed by 8 consecutive light flashes (1 second, followed by 29-second interstimulus dark intervals). Average responses represented as V1, first 2 seconds, and V2, the following 28 seconds. (**H**) SA and (**J**) average pattern of WT and *sucla2^–/–^* zebrafish larvae with and without *sirt5* overexpression treated with 250 μM of NAM/NR for 40 hours. (**I**–**L**) Quantification of mean activity per individual (WT, *n* = 18; *sucla2^–/–^*, *n* = 20; *sucla2^–/–^* + NAM/NR*, n* = 8; *sucla2^–/–^ sirt5^–/–^* + NAM/NR*,* *n* = 18, 7 dpf, fed). *P* values calculated by ordinary 1-way ANOVA with Tukey’s multiple-comparison test. Statistical analysis of survival curves (**A** and **E**) performed by log-rank test with Bonferroni’s correction. *P* values in **F** calculated by Fisher’s exact test. Box plots show median, first to third quartile, minima, maxima.

The execution of locomotor responses in response to a stimulus requires rapid activation of energy stores and resupply from mitochondria in neurons and muscle. Sirt5 and NAD^+^ supplementation restored basal and maximal bioenergetics capacity (Figure 4, G–I), suggesting that locomotor coupling could benefit from improved energy supply, and ultimately the overall health of *sucla2^–/–^* animals. We first performed survival time courses and found that lifespan was extended in *Tg(ubi:sirt5;cryaa:zsgreen) sucla2^–/–^* zebrafish compared with *sucla2^–/–^* zebrafish (Figure 6A). Next, we applied the LFR protocol that was used to characterize the progressive locomotor impairment of *sucla2^–/–^* carriers during larval development (Figure 2, G–I) and found that *Tg(ubi:sirt5;cryaa:zsgreen) sucla2^–/–^* animals showed an improved LFR compared with *sucla2^–/–^* zebrafish (maximum activity, *P* = 0.0278; number of events, *P* = 0.0093; *Tg(ubi:sirt5;cryaa:zsgreen) sucla2^–/–^* vs. *sucla2^–/–^;* 7 dpf) (Figure 6, B–D).

Supplementation with NAM/NR from 5 to 10 dpf showed a trend toward improved survival of *sucla2^–/–^* animals (*sucla2^–/–^* vs. *sucla2^–/–^* + 250 μM NAM/250 μM NR, 14-day time course, treated 4–10 dpf, *P* = 0.0824) and significant differences of animals alive at 14 dpf (*P* = 0.0171) (Figure 6, E and F). Next, we aimed to assess the effects of NAM/NR on locomotor phenotypes. Daily activities require repetitive neuronal and muscle functions, a capability that is frequently impaired in children affected by mitochondrial diseases. To address in greater detail the effects on phenotypes that resemble repetitive locomotor efforts, we extended the protocol to quantify responses to 8 consecutive light flashes in addition to sustained, spontaneous swimming behavior (Figure 6G). Notably, NAM/NR treatment improved baseline activity in the majority of *sucla2^–/–^* larvae (*sucla2^–/–^* vs. *sucla2^–/–^* + 250 μM NAM/250 μM NR; *P* = 0.0032) (Figure 6, H and I). In contrast, the responder rate was strongly blunted in *sucla2^–/–^ sirt5^–/–^* animals supplemented with NAM/NR (Figure 6, H and I). Similarly, NAM/NR-treated *sucla2^–/–^* larvae showed an improved LFR, integrating responses to the 8 repetitive stimuli (visual 1, V1) in addition to higher swimming activity in between light stimuli (visual 2, V2) (Figure 6, J–L). These effects were again lost in the additional background of genetic *sirt5* deficiency.

Thus, phenotypes that resemble locomotor defects in patients can be improved by restoration of intracellular NAD^+^ levels but require Sirt5 to protect from succinylation-induced damage.

## Discussion

Acyl-CoA species emerge as a group of endogenous reactive chemical entities that contribute to the progression of mitochondrial diseases. Here, we provide evidence that excess succinyl-CoA and bulk succinylation deplete NAD^+^ levels, thereby limiting its availability as a cofactor for Sirt5, with detrimental effects on mitochondrial bioenergetics and locomotion. Restoring NAD^+^ through supplementation with NR and NAM rescues mitochondrial respiratory failure in *sucla2^–/–^* zebrafish but not in the background of *sirt5* loss of function, indicating a surprising level of specificity. This mechanism of action suggests that succinylation-induced NAD^+^ depletion and impaired Sirt5 function are causal to mitochondrial respiratory defects in SCL deficiency. The effects of NAM/NR on spontaneous activity and the responsiveness to a light stimulus are effective when treatment is initiated at postembryonic stages after locomotor circuitry has developed. This finding is of potential therapeutic relevance as it suggests a time window to restore NAD^+^ and improve bioenergetics and urea elimination before irreversible damage is caused. NAD^+^ depletion has been proposed as a pathomechanism in other mitochondrial diseases (1, 44). Previous studies showed therapeutic effects of NR in mice carrying deletions in the gene encoding for the mitochondrial helicase *Twinkle*, which causes mitochondrial myopathy in humans (45). Importantly, a clinical study in a small number of adult patients carrying mutations in *TWINKLE* showed efficient restoration of NAD^+^ levels and improved muscle strength in response to daily supplementation with niacin (46). If NAD^+^ status becomes a clinically relevant marker for mitochondrial diseases that can be therapeutically targeted, the choice of the right NAD^+^ precursor is critical to optimize patient benefits. Niacin is a lipid-lowering drug that can cause skin flushing, potentially affecting compliance and largely restricting it to use in patients with very high serum lipids (47). NAD^+^ homeostasis is enabled through different metabolic pathways across organs, which affects how efficiently a precursor can increase NAD^+^ in the respective tissue. For example, the liver relies on NRK1 for efficient NAD^+^ homeostasis (48). In contrast, skeletal muscle NAD^+^ salvage is proposed to depend largely on the activity of NAM phosphoribosyltransferase (41, 49). The contribution of precursors to mitochondrial NAD^+^ homeostasis is also regulated by transport mechanisms (50). Here, we chose NR and NAM based on the efficient increase of NAD^+^ levels in WT and *sucla2^–/–^* larvae. However, we acknowledge that this approach may not be adequate to predict oral bioavailability and biodistribution in humans. Nevertheless, the finding that NAM complements NR efficiency is of interest: NAM at high concentrations has been shown to negatively affect sirtuin activity through product inhibition, including that of SIRT5, and is thought to not be a suitable NAD^+^ precursor in conditions where high levels of protein acylation occur (51, 52). However, although the effects of NAM on NAD^+^ levels in vivo are less studied compared with those of NR or NMN, NAM has been reported to have neuroprotective, nephroprotective, and cardioprotective effects in mice (53–55). Treatment of aged mice with NAM further protected from the formation of glaucoma (56, 57). Human studies have shown that oral NAM supplementation increases circulating levels of NAD^+^-related metabolites with promising signs to benefit acute kidney failure (54). In patients with mild-to-moderate COVID-19 infections, NAM accelerated recovery, further indicating oral availability and beneficial effects through NAD^+^ restoration in humans (58). Translational studies are warranted to explore the most effective combination of vitamin B3 forms on mitochondrial respiratory function in different tissues in mammalian models, and ultimately in patients. The remarkable specificity of NAM/NR supplementation for Sirt5 activation and improvement of phenotypes related to pathological succinylation seen in this study may not be directly translatable to other mitochondrial diseases. However, the mechanism shown here that an abundance of excess acyl-CoA activates endogenous sirtuin pools and therefore consumes NAD^+^ may be of broader relevance.

Our study provides insights into cellular signaling effects caused by protein acylation. We propose that protein succinylation triggers a rewiring of amino acid metabolism to enhance utilization of amino acids for oxidative metabolism. Consistent with this hypothesis, *sucla2^–/–^* animals deplete steady-state levels of glutamine and proline, two anaplerotic amino acids (37, 59). Findings in cell lines carrying mutations that lead to complex I deficiency show an essential requirement of glutamine catabolism in maintaining mitochondrial bioenergetics (60). If amino acids are an important carbon source for the generation of ATP in mitochondrial diseases, this process would generate excess nitrogen. Because nitrogen cannot be oxidized, it requires elimination through the urea cycle in the liver. Shuttling of nitrogen from peripheral organs occurs mainly in the form of alanine, a metabolic pathway termed the Cahill cycle (61). Hyperammonemia occurs if nitrogen is not efficiently excreted and causes neurotoxicity, for example, in inherited disorders of the urea cycle or in end-stage liver failure, but may also be relevant in mitochondrial diseases associated with high cellular acylation loads (62, 63).

The urea cycle is a known target of Sirt5, and the deacylation of CPS1 has been reported to be functionally relevant for efficient nitrogen elimination (17, 43). Although we did not observe differences in proteome-wide bulk succinylation in response to NAM/NR supplementation, we propose that CPS1 and potentially other enzymes of the urea cycle regain in function when NAD^+^ levels are increased and Sirt5 activity is restored. We cannot exclude, however, that normalized NAD^+^ levels improve SIRT5 functions other than its desuccinylase activity. Taken together, we propose that *sirt5* enables amino acid–derived oxidative metabolism by promoting urea excretion. A defect in excretion of nitrogen is indicated by elevated citrulline levels in *sucla2^–/–^* animals and therefore nitrogen fixation in the urea cycle. Sirt5 decreases citrulline levels while urea levels increase, suggesting a restoration of nitrogen flux by Sirt5. Supplementation with NAM/NR is sufficient to normalize urea generation in *sucla2^–/–^* animals relative to WT controls in a Sirt5-dependent manner. Studying ammonia and urea levels in children with SCL disease would be important to establish relevance for clinical applications.

Mitochondrial diseases are multisystem disorders that are difficult to study in vitro. Here, we describe behavioral phenotyping, including kinematic movement profiling, prey capture assays, and simplified LFR assays, to interrogate mechanisms of impaired coupling of neuronal stimuli to locomotor execution in a vertebrate disease model. Based on this paradigm, we identified restoration of NAD^+^ metabolism and reactivation of Sirt5 as a therapeutic avenue in SCL deficiency and potentially other conditions characterized by excess acyl-CoA.

## Methods

### Sex as a biological variable.

All experiments in this study were conducted with zebrafish larvae at stages prior to sex determination. This stage was chosen due to the unique advantages of monitoring for neurobehavior when the body plan is fully developed but the larvae are small enough for discovery of therapeutics. Potential sex-specific effects of the findings from this study require confirmation in suitable mammalian models and ultimately in human clinical studies.

### Antibodies and reagents.

Antibodies and reagents used in this study are shown in Supplemental Table 1.

### Zebrafish husbandry and generation of zebrafish lines.

Adult zebrafish of the commonly used AB line were raised at 28°C under standard husbandry conditions. Transgenic and mutant lines used in this study have been used in a previous publication (28). The transgenic line was registered at the central repository ZFIN.org under the designation *Tg(ubi:sirt5;cryaa:zsgreen1)^nei005^*. The transgenic line can be found on www.zfin.org in the section “Tg/Mutants” by entering the identifier (e.g., nei005) in the search field. The zebrafish mutant lines were registered at the central repository ZFIN.org with designations *sucla2^–/–nei010^* and *sirt5^–/–nei004^*. The mutant lines can be found on www.zfin.org in the section “Tg/Mutants” by entering the identifier (e.g., nei004) in the search field. A description of the methodology used to generate transgenic and mutant zebrafish lines is provided in the Supplemental Methods.

### Zebrafish larvae behavior profiling.

The behavior of each larva was recorded in an acrylic arena with a rounded edge, having a diameter of 50 mm and a depth of 4 mm. The rounded edge was achieved by a progressive bevel with a radius of curvature of 11 mm. Fish were imaged using an infrared array consisting of 64 LEDs of 850 nm (TSHG6400), a fixed focal length lens (Edmunds Optics, 86-207, f = 16 mm), an infrared long-pass filter (VisionLightTech, LP780-37, 780 nm), and a high-speed camera (Mikrotron EoSensCL, MC1362). Camera images were acquired using a frame grabber (National Instruments, PCIe-1433). For visual stimulation, a video projector (Optoma, ML750e) was used along with a neutral density filter (Thorlabs, NE06A) and a cold mirror (Edmunds Optics, 64-452) to project an image onto a diffuser screen (3 layers of Rosco Cinegel White Diffuser 3000) positioned 5 mm below the larva. For acoustic stimuli, a transducer speaker (VISATON, 4501) glued to the acrylic plate beneath the fish area was utilized. The quantification of movement categories and kinematic responses during the duration of the experiment was performed as described previously (32). A detailed description is provided in the Supplemental Methods.

The biostatistician quantifying zebrafish behavior was blinded to the genotypes, which were discriminated using HRM-based genotyping.

### Spontaneous activity quantification and light-evoked locomotion.

Larval spontaneous activity monitoring and light-flash response experiments were performed in zebrafish larvae using the ViewPoint ZebraBox system and the ZebraLab software (Viewpoint Life Sciences) that records the movement as “actinteg” units, which represent the sum of all pixel changes detected during the time slice defined for the experiment (1/15 of second). Two protocols were set up to measure larval spontaneous activity and LFR locomotion by using different light-dark transitions. Larvae were placed in a 96-well plate in 200 μL of egg water at indicated stages (1 larva per well). An initial simple protocol that alternated 5 minutes of dark for acclimatization followed by 10 seconds of light (100% intensity) was designed to assess LFR locomotor activity of *sucla2^–/–^* with or without *sirt5* overexpression. An extended protocol was used to measure consecutively different metrics: spontaneous activity, integrated LFR (V1), and integrated post-LFR (V2) (36) in WT and *sucla2^–/–^* zebrafish larvae with and without KO of *sirt5* background, immersed in egg water or treated with 250 μM of NAM/NR. After 5 minutes of adaptation in the dark, spontaneous activity was quantified for 10 minutes followed by 8 cycles alternating 1 second of light and 29 seconds of dark. This protocol was used to increase sensitivity of the assay. During the light phase of the simple LFR protocol, the light response was defined as the activity recorded in the first 4 seconds out of 10 because after that, the larvae entered a state of freezing with movements near zero. The maximal activity was calculated as the maximal amplitude of movement reached by the individual during the time period, and the number of events as the number of times a movement was detected for individuals. For the extended protocol, V1 was defined as the triggered average of the first 2 seconds after each light flash and V2 as the triggered average of the following 28 seconds in the dark. Raw data were then summarized and represented as mean activity per second or mean activity per individual. Larvae genotypes were distinguished by a fluorescence-based noninvasive method before the experiment and confirmed using the HRM method (LightCycler96, Roche Life Sciences). All analyses were conducted using R software (version R 4.1.1).

### Food intake determination.

Zebrafish larvae were raised under standard housing conditions and fed twice a day with dry food (Planktovie, GEMMA 75), starting at 5 dpf. At 7 dpf, larvae were collected 1 hour after the feeding cycle and anesthetized in a solution of 0.016% tricaine methanesulfonate diluted in egg water. Larvae were screened for food intake by examining the contents of their intestines using a binocular loupe. Experiments were conducted blindly as genotypes were discriminated using the HRM method a posteriori (LightCycler96, Roche Life Sciences).

### Alamar blue dye treatment and genotype discrimination.

Alamar blue (Thermo Fisher Scientific) working solution was prepared as follows: 0.04X Alamar dye (2× stock), 8 mM NaHCO^3^ (400 mM stock) and 0.2% DMSO (100% stock) diluted in egg water. Zebrafish larvae originating from a heterozygous in-cross were placed in black 96-well plates with a clear bottom (Greiner). One larva per well was distributed in 125 μL of egg water, and 75 μL of Alamar blue working solution was added to each well. The plate was incubated in the dark at 28.5°C for 18 hours, and fluorescence was recorded using a conventional plate reader (Flex Station3, Molecular Devices) at 544 nm of excitation and 590 nm of emission. To discriminate the different genotypes within the heterozygous in-cross, fluorescence values were plotted according to their intensity. Larvae with the corresponding fluorescence values above the 75th percentile (Q3) were sorted as homozygous mutants, and the ones with values under the 25th percentile (Q1) were sorted as WT animals and used for downstream assays. For experiments in which the noninvasive sorting approach was applied, independent crossings of WT AB lines were performed to generate WT controls. This approach was chosen to ensure WT controls because the pool of zebrafish with a low Alamar dye signal contained a mix of WT and heterozygous genetic backgrounds. The pool of animals with a high Alamar dye signal was selected to facilitate rapid experimentation with *sucla2^–/–^* animals.

### Prey capture assay.

For prey capture assays, the fish were fed with live rotifers (*Brachionus plicatilis*) as previously described (32). The behavioral setup employed in these experiments was similar to methods described in the zebrafish behavior profiling section but with a higher image resolution (365 pixels per cm) to enable tracking of the eyes and visualization of rotifers. A detailed description of the methodology based on the previous work to monitor zebrafish within the arena is outlined in the Supplemental Methods.

Behavioral quantification was performed using a MATLAB script (MathWorks). To compare the hunting behavior of the fish over time, larvae were grouped by genotype, and the average number of remaining rotifers was calculated for each frame. A median filter was applied over a 10-second rolling window to remove any anomalous counts that might come from small movements in the setup that disrupt the background model. Data were normalized to the number of rotifers detected over the first minute, and this number was subtracted from 1 to estimate the proportion of rotifers consumed. To assess differences in prey hunting among genotypes, a representative time point of 15 minutes was selected. This point corresponded to the time when the rate of capture for WT fish begins to slow down due to satiation. Hunting events were detected by using eye convergence because larval zebrafish converge their eyes when they detect and begin to attract prey. The starts and ends of hunting events were aligned with swim bouts, with the start of an event corresponding to a bout where the eyes become converged (defined as >60° of convergence) and the end corresponding to the end of the last bout before which the eyes are still converged. Comparisons were made of the number and length of hunting events in 5-minute intervals over the 15-minute period. Differences between mutant and WT sibling values were tested using a nonparametric Mann-Whitney *U* test (2-tailed).

### Zebrafish biometrics.

Whole larvae were anesthetized and aligned in a mixture of 0.016% tricaine and 3% methylcellulose. Brightfield images were then captured using a Leica M165FC stereo microscope with LAS X software, allowing for the measurement of larval length and muscle diameter using Qupath (version 0.5.1). Prior to the experiment, genotypes were distinguished using the Alamar method, and then the larvae genotypes were confirmed through the HRM method. WT larvae were obtained from an independent AB crossing.

### Phalloidin staining and imaging.

Zebrafish larvae were fixed in 4% paraformaldehyde for 2 hours at room temperature. After 3 washes in PBS containing 0.1% Tween 20, the larvae were permeabilized for 90 minutes in PBS with 2% Triton X-100 under agitation. The larvae were then stained overnight at 4°C with Alexa Fluor 546 Phalloidin (Thermo Fisher Scientific, A22283) diluted 1:20 in PBS with 2% Triton X-100. After staining, the larvae were washed in PBS and embedded in 1.5% low melting agarose to a 96-well black plate with a transparent glass bottom for visualization using a 20× objective. Images were acquired using a Leica sp8 microscope. The resolution achieved was 561 nm per pixel. Image stacks were acquired every 4 μm. Images were processed in ImageJ (NIH), and only linear modifications to brightness/contrast were made.

### Total protein isolation and immunoblotting.

Total proteins were extracted from zebrafish larvae (pool of ~16 to 20 whole larvae) with RIPA buffer (Sigma-Aldrich) supplemented with protease inhibitor cocktail (Thermo Fisher Scientific) using a tissue homogenizer (TissueLyserII, QIAGEN). Protein quantification was performed using a BCA assay kit (Thermo Fisher Scientific). Next, 20 μg of proteins were loaded onto precast NuPAGE Bis-Tris gels 4%–12% (Thermo Fisher Scientific) and transferred onto nitrocellulose membranes using the Trans-Blot Turbo Transfer System (Bio-Rad). Pan anti-succinyl-lysine (PTM Biolabs, PTM-401), Sucla2 (Abcam, ab183513), Hsc-70 (Santa Cruz Biotechnology, sc-7298), tubulin (Abcam, ab6046), and Vdac1 (Abcam, ab15895) were used as primary antibodies. Anti-rabbit and anti-mouse IgG HRP-labeled antibodies (PerkinElmer) were used as secondary antibodies.

A full list of antibodies and dilution factors used are specified in Supplemental Table 1. Uncropped Western blot images are shown in the Source Data file and in the supplemental materials. Visible bands at the molecular weight of sucla2 antibodies are due to the presence of small amounts of protein from *sucla2^+/–^* animals among the predicted *sucla2^–/–^* genotypes based on the sorting protocol presented in Figure 2.

### Biological markers: urea, NAD^+^, and lactate measurements.

For biological markers, larvae were pooled in lysis buffers and homogenized using 5 mm stainless steel beads in a tissue lyser (QIAGEN) for 2 minutes at 30 Hz. Urea and NAD^+^ were extracted from larvae homogenate (pool of minimum 12 larvae) and quantified using a Urea Colorimetric assay kit (Biovision) and an EnzyChrom NAD/NADH assay kit (BioAssay Systems), respectively, according to the manufacturer’s instructions. Lactate concentrations were determined by a fluorescent enzymatic assay. Samples (pool of 11 larvae) were homogenized and diluted in assay reagent containing 100 mM sodium phosphate (pH 7.5), 0.1 mM EDTA, 0.05 mM Amplex UltraRed, 0.1 U/mL lactate oxidase, and 1.5 U/mL HRP. Fluorescence was recorded after 30 minutes using a conventional plate reader (Flex Station3, Molecular Devices) at 500 nm of excitation and 600 nm of emission. Larvae for this experiment were discriminated by the fluorescence-based noninvasive method presented in Figure 2 and selected for downstream experimentation.

### Oxygen consumption rate measurements.

Zebrafish larvae were placed in wells of a 24-well islet microplate (1 larva in 600 mL of egg water per well) and maintained with an islet capture screen. The plate was loaded into a Seahorse XF24 Extracellular Flux Analyzer (Agilent), and the oxygen consumption rate was measured every 5 minutes. Basal respiration was calculated using the average of the last 3 points measured before the injection of 2 μM carbonyl cyanide-p-trifluoromethoxyphenylhydrazone (FCCP) to permit the electron transport chain to function at its maximal rate. Uncoupling respiration was determined by the maximal value after FCCP injection. Finally, 1 μM rotenone and 1 μg/ml antimycin A were injected to inhibit the electron transport chain and reveal nonmitochondrial respiration, which was deducted from both basal and uncoupling respiration values. Temperature was kept at 28.5°C during the whole experiment. The experiment was repeated 3 times in the exact same conditions to ensure reproducibility and solidity. Larvae for this experiment were discriminated by the fluorescence-based noninvasive method presented in Figure 2 and selected for downstream experimentation.

### Identification and quantification of metabolites.

Intracellular metabolites from zebrafish were harvested from pools of 10 larvae that were snap-frozen in liquid nitrogen and kept at –80°C before analysis. A cold biphasic extraction was employed to obtain dried samples. A detailed description of sample purification steps is in the Supplemental Methods.

Five microliters of dry samples were injected into a Vanquish UHPLC (Thermo Fisher Scientific), equipped with a hydrophilic liquid chromatography column (ZIC-pHILIC column, 100 × 2.1 mm, 5 μm, with a ZIC-pHILIC guard column, 20 × 2.1 mm, 5 μm, both from Merck SeQuant). Separation was achieved by applying a linear solvent gradient in decreasing organic solvent (90%–25%, 15.5 minutes) at 0.2 mL/min flow rate and 35°C. Aqueous 10 mM ammonium acetate with 0.04% (v/v) ammonium hydroxide (A) and acetonitrile (B) were used as mobile phases. The eluting metabolites were analyzed on an Orbitrap Fusion Lumos mass spectrometer (Thermo Fisher Scientific) with a heated electrospray ionization (H-ESI) source. The mass of the metabolites was assessed with on-the-fly positive and negative mode switching at a resolution of 60,000 at an m/z of 200. The spray voltages were 3,500 V and 3,000 V for positive and negative modes, respectively. The sheath gas was 20 AU, and the auxiliary gas was kept at 15 AU. The temperature of the vaporizer was 280°C, and the temperature of the ion transfer tube was 310°C. Instrument control and peak integration were conducted with Xcalibur 4.2.47 software (Thermo Fisher Scientific). Metabolites were identified according to their exact mass, and the signal intensities were normalized to a ^13^C internal standard.

Larvae for this experiment were discriminated by the fluorescence-based noninvasive method presented in Figure 2 and selected for downstream experimentation.

### NAD^+^ precursor treatments.

NAD^+^ precursors were administered to the animals at 5 dpf by direct immersion in egg water supplemented with 10 mM HEPES as a buffering agent. Single NAD^+^ precursors were used at a final concentration of 500 μM, and a combination of NR and NAM was used at a final concentration of 250 μM for each of the compounds. Treatments were performed for 40 hours from 5 to 7 dpf for the majority of experimental readouts and for 5 days from 5 to 10 dpf for survival experiments.

### Zebrafish survival assays.

*Sucla2^–/–^* and WT zebrafish larvae with or without *sirt5* overexpression were transferred at 5 dpf into 1.1 liter tanks and kept under constant water flow with access to food (Planktovie, GEMMA 75, twice a day) for 48 hours. At 7 dpf, animals were transferred into 96-well plates in a volume of 200 μL of egg water and survival was monitored frequently for 130 hours onward without any exchange of water, allowing a progressive acidification of the medium. In a second assay, *Sucla2^–/–^* and WT zebrafish larvae with and without KO of *sirt5* were transferred at 5 dpf into 3.5 liter tanks with access to food during the whole experiment (Planktovie, GEMMA 75, twice a day). From 5 to 10 dpf, animals were treated with 250 μM of NAM/NR combination or egg water for the control groups. Treatment was replenished daily. Survival was monitored twice a day from 5 dpf to 14 dpf. Larvae were counted as dead if they were found dead in the well or terminally ill. This was defined as clearly visible signs not compatible with survival such as cardiac edemas and inability to respond to touch with a tail movement. Larvae for this experiment were discriminated by the fluorescence-based noninvasive method presented in Figure 2 and selected for downstream experimentation.

### Statistics.

Statistical analyses were performed as indicated in the main text and figure legends and included the 2-tailed *t* test, nonparametric Kruskal-Wallis test, and Fisher’s exact test. Longitudinal data of zebrafish larvae were analyzed with Kaplan-Meier survival curves with pairwise comparison using a log-rank test. *P* value adjustment was performed using Bonferroni’s post hoc test. LFR data representing the numbers of events and maximal activity were analyzed by using a nonparametric test (Kruskal-Wallis) with Dunn’s multiple-comparison test. Box plot elements show the following data: center line, median; box limits, upper and lower quartiles; whiskers, 1.5× IQR; points, outliers. *P* values of less than 0.05 were considered significant.

### Study approval.

Experimental procedures were carried out according to Swiss and EU ethical guidelines (European Directive 2010/63/EU). The experiments were approved by the animal experimentation ethical committee of Canton of Vaud (permits VD-H13 and VD3177/VD3546), the Champalimaud Foundation Animal Welfare Body (ORBEA), and the Portuguese Direcçao Geral Veterinária.

### Data availability.

R-scripts used in this study to monitor LFR and to quantify free swimming larvae in the behavior arena have been deposited in GitHub and can be accessed through the following links: LFR (https://github.com/giulializzo/Zebrafish_Zebrabox_Activity_Analysis) and prey capture assay (https://github.com/orger-lab/sucla_preycapture; free swimming analysis, https://github.com/orger-lab/megabouts). Metabolomics data underlying the analysis of metabolite changes in WT and *sucla2^–/–^* larvae are provided in Supplemental Data File 1. For the source data, the Supporting Data Values file is available as part of the supplemental materials.

## Author contributions

PG conceived the study, supervised the work, and wrote the manuscript. JR designed, performed, and analyzed experiments and contributed to the writing of the manuscript. AJ and PTMS performed experiments, data analysis, and interpretation of zebrafish behavior profiling under the supervision of MBO. GL wrote the scripts for the LFR workflow, helped with the experimental design of the LFR, and contributed to statistical analyses. SM and SC performed metabolomics experiments and data analyses. NR and AP contributed to the design and execution of experiments. All authors read and commented on the manuscript.

## Funding support

MBO was supported by FCT (Portugal; PTDC/MED-NEU/32664/2017 financed by Lisboa2020, project LISBOA-01-0145-FEDER-036264), Volkswagen Stiftung, and ERC (NEUROFISH 773012).

- The Champalimaud Fish Facility was supported by Congento LISBOA-01-0145-FEDER-022170, cofinanced by FCT (Portugal) and Lisboa2020, under the PORTUGAL2020 agreement (European Regional Development Fund).
- AJ was supported by “la Caixa” Foundation (LCF/BQ/PR20/11770007).

## Supplementary Material

Supplemental data

Supplemental data set 1

Unedited blot and gel images

Supporting data values

## Version 1 01/23/2026

Electronic publication

## References

[1] Russell OM (2020). Mitochondrial diseases: hope for the future. Cell.

[2] Vafai SB, Mootha VK (2012). Mitochondrial disorders as windows into an ancient organelle. Nature.

[3] Gorman GS (2016). Mitochondrial diseases. Nat Rev Dis Primers.

[4] McFarland R (2010). A neurological perspective on mitochondrial disease. Lancet Neurol.

[5] Ylikallio E, Suomalainen A (2012). Mechanisms of mitochondrial diseases. Ann Med.

[6] Sheu SS (2006). Targeting antioxidants to mitochondria: a new therapeutic direction. Biochim Biophys Acta.

[7] Trub AG, Hirschey MD (2018). Reactive Acyl-CoA Species modify proteins and induce carbon stress. Trends Biochem Sci.

[8] Wagner GR, Hirschey MD (2014). Nonenzymatic protein acylation as a carbon stress regulated by sirtuin deacylases. Mol Cell.

[9] Wagner GR (2017). A class of reactive acyl-CoA species reveals the non-enzymatic origins of protein acylation. Cell Metab.

[10] James AM (2018). The causes and consequences of nonenzymatic protein acylation. Trends Biochem Sci.

[11] Choudhary C (2014). The growing landscape of lysine acetylation links metabolism and cell signalling. Nat Rev Mol Cell Biol.

[12] He W (2012). Mitochondrial sirtuins: regulators of protein acylation and metabolism. Trends Endocrinol Metab.

[13] Verdin E, Ott M (2015). 50 years of protein acetylation: from gene regulation to epigenetics, metabolism and beyond. Nat Rev Mol Cell Biol.

[14] Wagner GR, Payne RM (2013). Widespread and enzyme-independent Nε-acetylation and Nε-succinylation of proteins in the chemical conditions of the mitochondrial matrix. J Biol Chem.

[15] Weinert BT (2013). Lysine succinylation is a frequently occurring modification in prokaryotes and eukaryotes and extensively overlaps with acetylation. Cell Rep.

[16] Meyer JG (2016). Quantification of lysine acetylation and succinylation stoichiometry in proteins using mass spectrometric data-independent acquisitions (SWATH). J Am Soc Mass Spectrom.

[17] Rardin MJ (2013). SIRT5 regulates the mitochondrial lysine succinylome and metabolic networks. Cell Metab.

[18] Weinert BT (2015). Analysis of acetylation stoichiometry suggests that SIRT3 repairs nonenzymatic acetylation lesions. EMBO J.

[19] Gut P (2020). SUCLA2 mutations cause global protein succinylation contributing to the pathomechanism of a hereditary mitochondrial disease. Nat Commun.

[20] Carrozzo R (2016). Succinate-CoA ligase deficiency due to mutations in SUCLA2 and SUCLG1: phenotype and genotype correlations in 71 patients. J Inherit Metab Dis.

[21] Matilainen S, Isohanni P, Euro L, Lonnqvist T, Pihko H, Kivela T (2015). Mitochondrial encephalomyopathy and retinoblastoma explained by compound heterozygosity of SUCLA2 point mutation and 13q14 deletion. Eur J Hum Genet.

[22] Morava E (2009). Dystonia and deafness due to SUCLA2 defect; Clinical course and biochemical markers in 16 children. Mitochondrion.

[23] Carrozzo R (2007). SUCLA2 mutations are associated with mild methylmalonic aciduria, Leigh-like encephalomyopathy, dystonia and deafness. Brain.

[24] Bruni G (2016). Zebrafish behavioral profiling identifies multitarget antipsychotic-like compounds. Nat Chem Biol.

[25] Baier H, Scott EK (2009). Genetic and optical targeting of neural circuits and behavior--zebrafish in the spotlight. Curr Opin Neurobiol.

[26] Pantoja C (2016). Neuromodulatory regulation of behavioral individuality in zebrafish. Neuron.

[27] Portugues R, Engert F (2009). The neural basis of visual behaviors in the larval zebrafish. Curr Opin Neurobiol.

[28] Douglass AD (2008). Escape behavior elicited by single, channelrhodopsin-2-evoked spikes in zebrafish somatosensory neurons. Curr Biol.

[29] Temizer I (2015). A Visual Pathway for Looming-Evoked Escape in Larval Zebrafish. Curr Biol.

[30] Burgess HA, Granato M (2007). Modulation of locomotor activity in larval zebrafish during light adaptation. J Exp Biol.

[31] Dunn TW (2016). Neural circuits underlying visually evoked escapes in larval zebrafish. Neuron.

[32] Marques JC (2018). Structure of the zebrafish locomotor repertoire revealed with unsupervised behavioral clustering. Curr Biol.

[33] Titov DV (2016). Complementation of mitochondrial electron transport chain by manipulation of the NAD^+^/NADH ratio. Science.

[34] Gut P (2017). Little fish, big data: zebrafish as a model for cardiovascular and metabolic disease. Physiol Rev.

[35] Gut P (2013). Whole-organism screening for gluconeogenesis identifies activators of fasting metabolism. Nat Chem Biol.

[36] Jordi J (2018). High-throughput screening for selective appetite modulators: a multibehavioral and translational drug discovery strategy. Sci Adv.

[37] Owen OE (2002). The key role of anaplerosis and cataplerosis for citric acid cycle function. J Biol Chem.

[38] Anderson KA (2017). Metabolic control by sirtuins and other enzymes that sense NAD^+^, NADH, or their ratio. Biochim Biophys Acta Bioenerg.

[39] Feldman JL (2015). Kinetic and structural basis for acyl-group selectivity and NAD(+) dependence in sirtuin-catalyzed deacylation. Biochemistry.

[40] Verdin E (2015). NAD^+^ in aging, metabolism, and neurodegeneration. Science.

[41] Liu L (2018). Quantitative analysis of NAD synthesis-breakdown fluxes. Cell Metab.

[42] Tan M (2014). Lysine glutarylation is a protein posttranslational modification regulated by SIRT5. Cell Metab.

[43] Nakagawa T (2009). SIRT5 Deacetylates carbamoyl phosphate synthetase 1 and regulates the urea cycle. Cell.

[44] Lightowlers RN, Chrzanowska-Lightowlers ZM (2014). Salvaging hope: is increasing NAD(+) a key to treating mitochondrial myopathy?. EMBO Mol Med.

[45] Khan NA (2014). Effective treatment of mitochondrial myopathy by nicotinamide riboside, a vitamin B3. EMBO Mol Med.

[46] Pirinen E (2020). Niacin cures systemic NAD^+^ deficiency and improves muscle performance in adult-onset mitochondrial myopathy. Cell Metab.

[47] Benyo Z (2005). GPR109A (PUMA-G/HM74A) mediates nicotinic acid-induced flushing. J Clin Invest.

[48] Ratajczak J (2016). NRK1 controls nicotinamide mononucleotide and nicotinamide riboside metabolism in mammalian cells. Nat Commun.

[49] Frederick DW (2016). Loss of NAD Homeostasis Leads to Progressive and Reversible Degeneration of Skeletal Muscle. Cell Metab.

[50] Davila A (2018). Nicotinamide adenine dinucleotide is transported into mammalian mitochondria. Elife.

[51] Fischer F (2012). Sirt5 deacylation activities show differential sensitivities to nicotinamide inhibition. PLoS One.

[52] Avalos JL (2005). Mechanism of sirtuin inhibition by nicotinamide: altering the NAD(+) cosubstrate specificity of a Sir2 enzyme. Mol Cell.

[53] Fricker RA (2018). The influence of nicotinamide on health and disease in the central nervous system. Int J Tryptophan Res.

[54] Poyan Mehr A (2018). De novo NAD^+^ biosynthetic impairment in acute kidney injury in humans. Nat Med.

[55] Abdellatif M (2021). Nicotinamide for the treatment of heart failure with preserved ejection fraction. Sci Transl Med.

[56] Williams PA (2017). Vitamin B_3_ modulates mitochondrial vulnerability and prevents glaucoma in aged mice. Science.

[57] Williams PA (2017). Glaucoma as a metabolic optic neuropathy: making the case for nicotinamide treatment in glaucoma. J Glaucoma.

[58] Schreiber S (2025). Nicotinamide modulates gut microbial metabolic potential and accelerates recovery in mild-to-moderate COVID-19. Nat Metab.

[59] DeBerardinis RJ (2008). The biology of cancer: metabolic reprogramming fuels cell growth and proliferation. Cell Metab.

[60] Barrow JJ (2016). Bromodomain inhibitors correct bioenergetic deficiency caused by mitochondrial disease complex I mutations. Mol Cell.

[61] White PJ (2020). Muscle-liver trafficking of BCAA-derived nitrogen underlies obesity-related glycine depletion. Cell Rep.

[62] Cagnon L, Braissant O (2007). Hyperammonemia-induced toxicity for the developing central nervous system. Brain Res Rev.

[63] Elwir S, Rahimi RS (2017). Hepatic encephalopathy: an update on the pathophysiology and therapeutic options. J Clin Transl Hepatol.

