# Supplementary material for: NAD+ and Sirt5 restore mitochondrial bioenergetics failure and improve locomotor defects caused by sucla2 mutations
Source: JCI Insight. 2026 Jan 23;11(2):e181812. doi: 10.1172/jci.insight.181812 (PMC12892911; doi:10.1172/jci.insight.181812)
Supplement: Supplemental data [file jciinsight-11-181812-s234.pdf]

SUPPLEMENTARY INFORMATION

NAD$^+$ and Sirt5 restore mitochondrial bioenergetics failure and improve locomotor defects caused by *sucla2* mutations

Joy Richard et al.

Supplementary Figures 1-4

Supplementary Methods

Supplementary Table 1

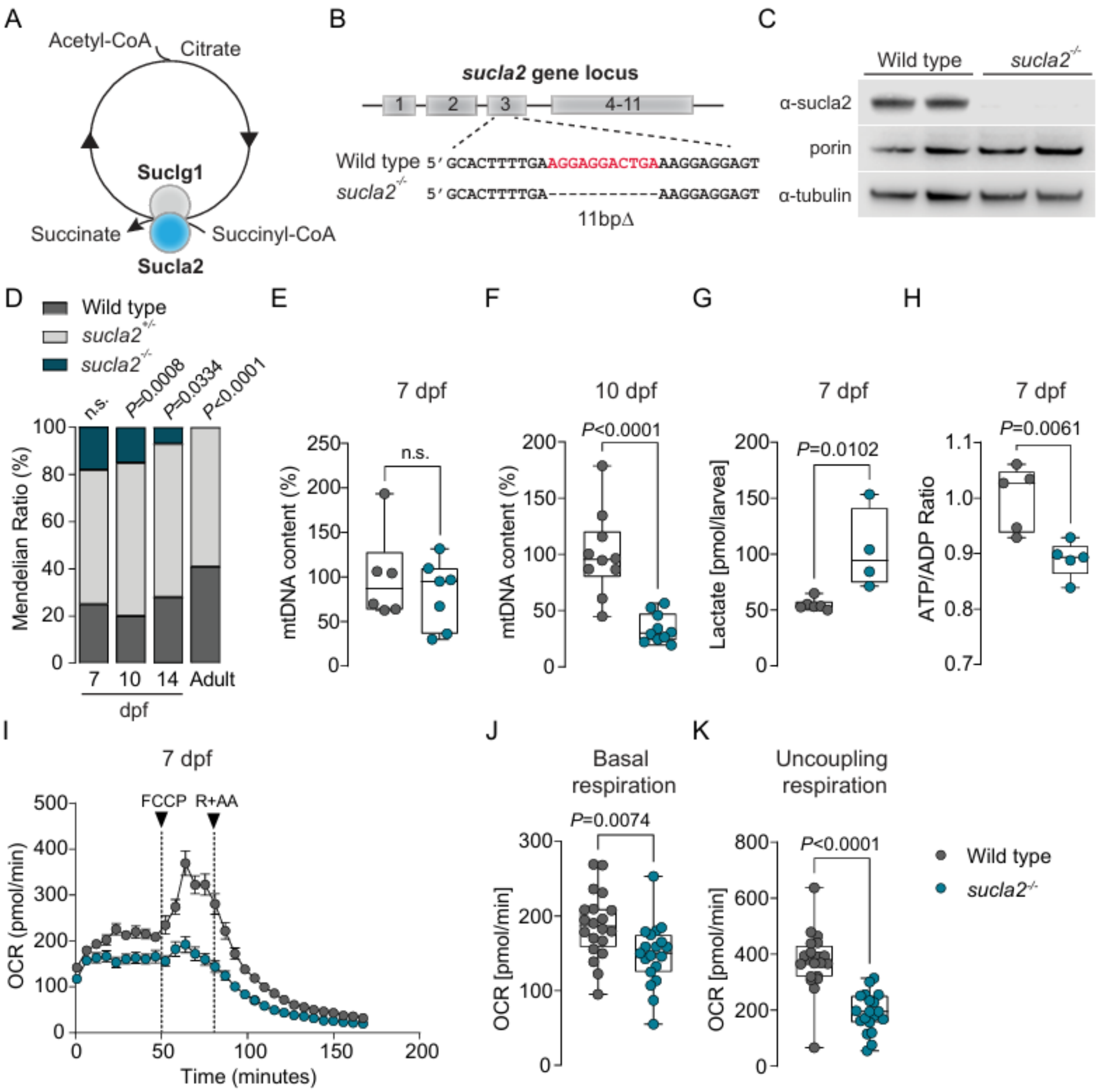

Supplementary Figure 1. ***sucla2-/-*** **zebrafish recapitulate hallmarks of human SCL deficiency**

A Schematic representation of the TCA cycle and enzymatic reaction of SCL. **B** Sequence alignments showing the introduction of an 11 base pair deletion on the exon 3 within the *sucla2* gene. **C** Western blot analysis of the sucla2 protein in zebrafish larvae with homozyogous mutations in *sucla2* and in control larvae (10dpf, pools of 28 dissected trunks). Tubulin and porin were used as cytosolic and mitochondrial marker proteins respectively. **D** Mendelian ratios obtained from incrosses of *sucla2* heterozygous animals genotyped at different stages of

development: 7dpf (n=200), 10dpf (n=156), 14 dpf (n=40) and at 5 months (n=56). Statistics correspond to p-values comparing observed versus expected distribution based on the genotype (Chi-square test) **E, F** qPCR analysis of mitochondrial DNA content in *sucla2*$^{-/-}$ and control zebrafish larvae at 7dpf (Wild-type, n=6; *sucla2*$^{-/-}$, n=7) and 10dpf (n=10). Results are represented in percentage normalized to Wild-type. **G** Lactate levels measurement from wild-type and *sucla2*$^{-/-}$ animals (Wild-type, n=6; *sucla2*$^{-/-}$, n=4; Pool of 11 larvae, 7dpf). **H** Metabolomics analyses of *sucla2*$^{-/-}$ and control zebrafish larvae showing ATP/ADP (Pool of 8 larvae; n=5; 7dpf). **I** Oxygen consumption rate (OCR) in *sucla2*$^{-/-}$ and control zebrafish larvae (Wild-type, n=20; *sucla2*$^{-/-}$, n=19; 7dpf), before and after the addition of inhibitors of mitochondrial respiration. The uncoupler FCCP is added to stimulate maximal OCR. Rotenone and antimycin A (R+AA) determine non-mitochondrial respiration. **J, K** Basal OCR calculating the average of the three last points before addition of FCCP (**J**) and maximal OCR (**K**). Genotypes represented in **I-K** were discriminated using fluorescence based non-invasive method. Statistics represented in **E-K** were calculated by standard t-tests. The boxplots show the median, the first to third quartile, minima and maxima. n.s. not significant. Source data are provided as a source data file.

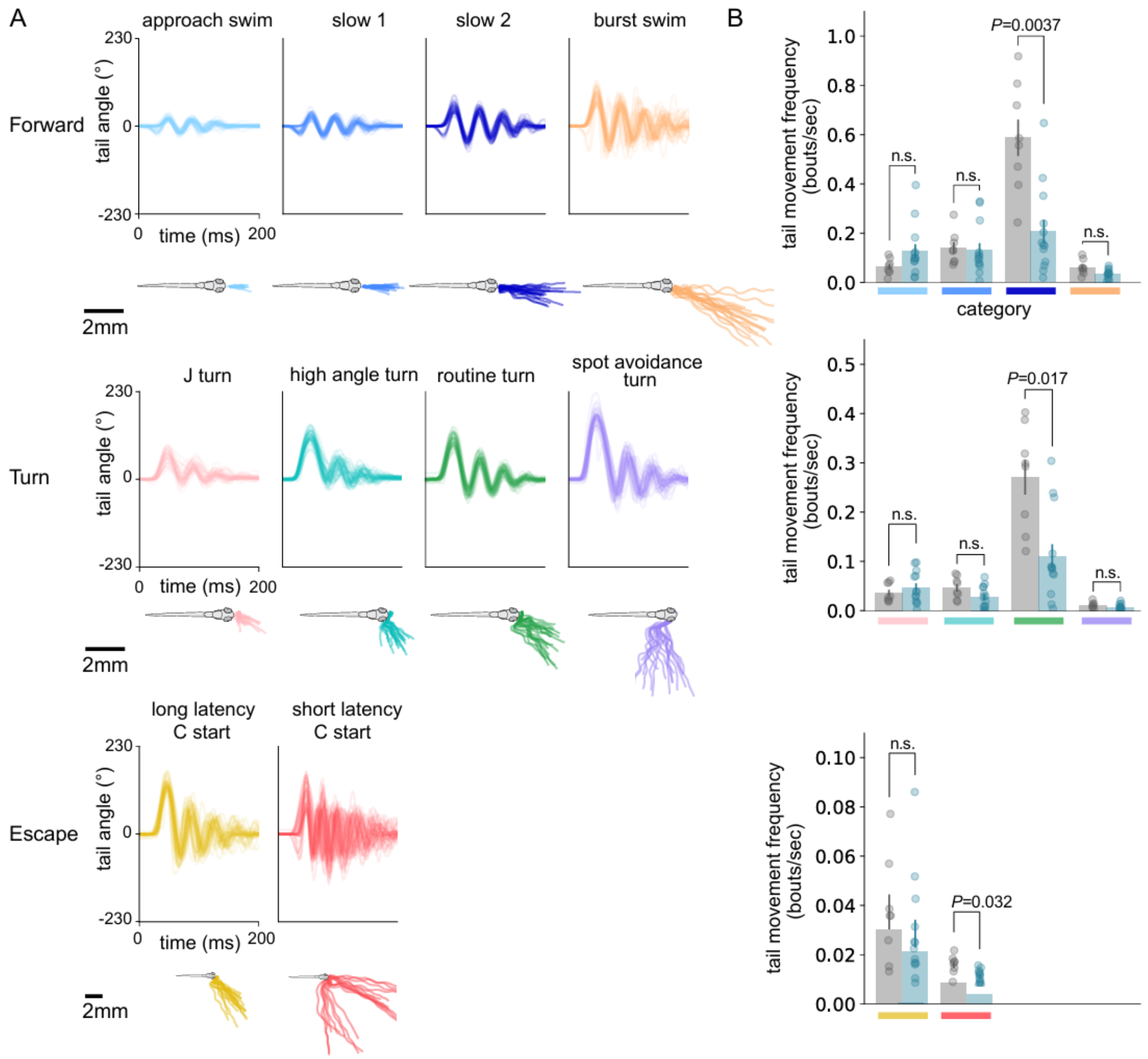

Supplementary Figure 2. Illustration of the locomotor repertoire profiling

A Illustration of the locomotor repertoire consisting of 10 tail movement categories in wild-type larvae: approach swim, slow1, slow2, burst swim, J turn, high angle turn, routine turn, spot avoidance turn, long latency C start, short latency C start. Tail angle and corresponding trajectories traces from 50 example bouts from wild type larvae (5-7dpf) are shown for each category. **B** Frequency distribution of each movement category of larvae of both genotypes (Wild-type, n=8; *sucla2*$^{-/-}$, n=13. 5-7 dpf). Statistics represented were calculated by standard t-tests with Bonferroni correction. n.s. not significant.

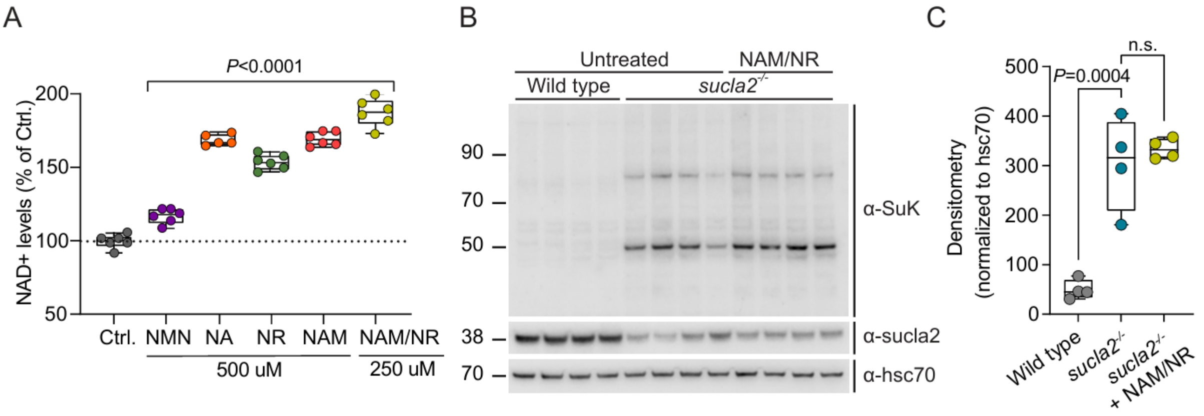

Supplementary Figure 3. Effects of NAM/NR treatment on NAD^+ levels and global protein succinylation

A NAD^+ levels measurement from 7dpf wild-type animals immersed in buffered egg water (untreated control) and in either 500 µM of different NAD+ precursors or in 250 µM of NR and NAM combination treatment during 40h (n=6 per group; Pool of minimum 13 larvae). Data are represented as a percentage of untreated control animals. **B** SDS-page and western blot analysis of global protein succinylation in 7dpf wild-type and *sucla2*$^{-/-}$ animals immersed either in buffered egg water (untreated) or in 250 µM of NR and NAM combination treatment during 40h (n=4; pools of 20 larvae). Pan-succinyl-lysine antibody was used to quantify lysine succinylation, and Hsc70 antibody was used as loading controls. The visible bands at the molecular weight of sucla2 antibodies is due to the presence of small amounts of protein from *sucla2*$^{+/-}$ animals among the predicted *sucla2*$^{-/-}$ genotypes based on the sorting protocol presented in **Figure 2**. Boxplot in **C** shows the quantification of the pan-succinyl-lysine western blots by Image J using the mean of the densitometry of the two main bands, normalized to Hsc70. Statistics correspond to p-values calculated by ordinary one-way Anova with multiple comparisons tests (Tukey). The boxplots show the median, the first to third quartile, minima and maxima. n.s., not significant. Source data are provided as a Source Data file.

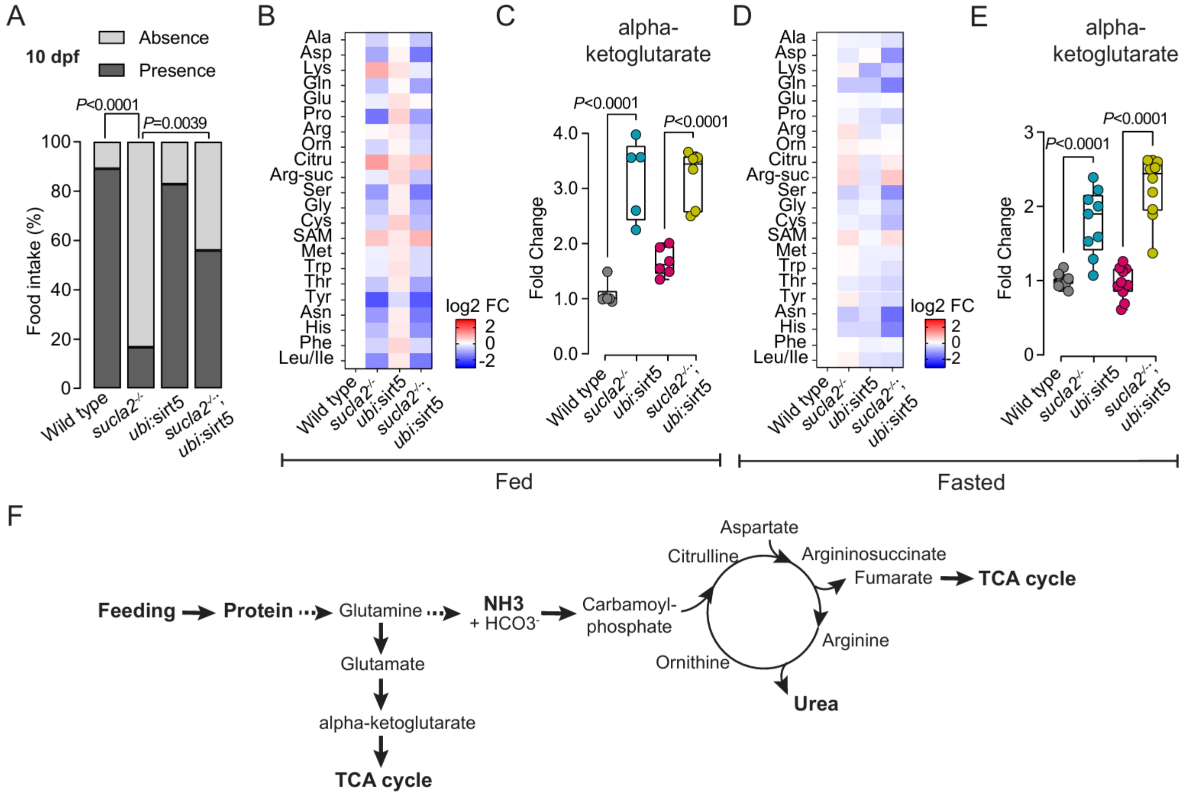

Supplementary Figure 4. Schematic of urea elimination

A Quantification of food intake in 10dpf *sucla2*$^{-/-}$ and control zebrafish larvae with or without *sirt5* overexpression (Wild-type, n=46; *sucla2*$^{-/-}$, n=30; *ubi:*sirt5, n=41; *sucla2*$^{-/-}$*;ubi:*sirt5, n=25). **B, D** Heatmap of relative abundance of amino acids in wild-type and *sucla2*$^{-/-}$ animals with and without overexpression of *sirt5* in (**B**) fed or in (**D**) fasted conditions (expressed as fold change relative to the median of wild-type controls). **C, E** Boxplots showing fold changes levels of alpha-ketoglutarate (**C**) fed or in (**E**) fasted conditions. **F** Schematic representation of amino acid catabolism through TCA cycle and link to nitrogen elimination by the urea cycle.

Supplementary Methods

Generation of genetically modified zebrafish lines

Transgenic zebrafish were generated using I-SCEI meganuclease-mediated insertion into AB embryos at the one-cell stage of a construct harboring the zebrafish *sirt5* under the control of the ubiquitous *ubi* promoter. For rapid selection of transgenic animals, the injected constructs carried an eye-marker cassette harboring *ZsGreen* under the control of the *cryaa* (alpha-crystallin A chain) promoter in reverse direction. Transgenic carriers were outcrossed with AB fish to raise transgenic and wild-type siblings. Homozygous animals were selected and incrossed and the progeny were used for experiments. Gene disruptions of the *sirt5* and *sucla2* loci in zebrafish were generated using CRISPR/Cas9 genome editing. Embryos were injected at the one-cell stage with recombinant CAS9 protein and single guide RNAs targeting exon 3 of *sucla2* and exon 5 of *sirt5*. Primers were designed to amplify a short amplicon flanking the targeted sites, and high-resolution melt (HRM) analysis was performed on genomic DNA of the injected embryos to confirm successful gene disruption using a SYBR PCR mix on a LightCycler96 (Roche Life Science). Primer pair sequences were used as follows: sucla2: 5′-CTTGGTTATAAAGCCCAAGTGC-3′ (forward) and 5′-GAGTAAACGATTCTGACTCCTCC-3′ (reverse); sirt5: 5′-GGGTGGGTAATTGGGAAGTT-3′ (forward) and 5′- GATGGTCCAGTCCTGGTTTG-3′ (reverse). Adult F0 zebrafish were outcrossed to wild-type AB animals to generate a F1 generation. HRM analysis on genomic DNA from tails of zebrafish adults was used to identify founder animals, and the out-of-frame mutations were confirmed using gene sequencing.

Evaluation of expected and real mendelian Ratio

Zebrafish larvae obtained from heterozygous incrosses were raised under normal feeding conditions. Lethality within the incrosses was assessed at different stages of development. DNA from larvae or adult zebrafish was extracted and their genotype was determined by high-resolution melting qPCR.

Behavioral profiling

Movement categories and kinematic responses were quantified as follows: the total duration of the experiments is 90 minutes. The term "virtual open loop" describes a situation in which the location of the visual stimulus is continuously updated (in an experimental closed loop) according to the current position and orientation of the fish. In this condition, the swimming of the zebrafish does not affect the relative position of the visual stimuli, which means the animal is in an open loop with respect to the stimulus. The sequences consisted of the following stimuli: Approaching dot; a black disk (1 mm radius), initially positioned 2 cm away from the fish, approaches at a speed of 5 mm/s. After the approach, the disk remains below the fish for 1 second. The direction of the disk is ± 90° away from the direction of the fish. This stimulus is displayed in a virtual open loop. Directional optomotor response; a moving grating with a spatial period of 10 mm moves at 10 mm/s at an angle of either ±45° or ±135° relative to the heading of the fish. This stimulus is displayed in a virtual open loop. Acoustic startle; a 100 ms pulse of 100 Hz. Forward optomotor response; a moving grating with a spatial period of 10 mm and a velocity of 8,16 or 24 mm/s displayed along the fish axis. For this stimulus, the orientation, but not the position of the grating was updated with the zebrafish's movements. Therefore, the animal could not change the relative direction of the grating by turning, but it could 'keep up' with the motion of the grating by swimming forwards. Light/Dark; the projection was turned to full black for a period of 30 seconds. All visual stimuli were programmed using the OpenGL Shading Language. The posture was computed in real-time using a C# program. It follows the tail curve using 10 segments, starting at the swim bladder.

Tail bouts were classified as previously described (1). It starts by smoothing the time series of tail angles over time. The onset and offset of tail motion are then determined using a threshold on the cumulative absolute value of the tail angles over the segments. For each tail bout, the kinematic parameters described previously (1) are computed. The kinematic parameters are projected onto 20 principal components to obtain the final feature vectors. Finally, the category of each movement is computed using a k-nearest neighbors classifier (k=50) from a predefined library of bouts; capture swim and O bend from the library were excluded because the dataset did not include prey capture or a sufficient number of O Bend (<100 across larvae). The vigor of tail bouts was computed as the total variance of the tail angle over time and tail segments. To compute the mobility, we take the derivative of the sum of the cumulative fish displacement in both trajectory (mm) and rotation (rad). A rolling window of 100 sec was used as long timescale estimation for the calculation of the derivative.

Prey Capture Assay

Monitoring of free swimming larvae in the arena was performed as follows: A Schneider apo-Xenoplan 2.0/35 lens (Jos. Schneider Optische Werke GmbH, Germany) was used for a reduced field of view. A diffuser screen consisting of three layers of Rosco Cinegel White Diffuser 3000 was positioned 5cm above the infrared LED array used for imaging and 9cm below the arena, providing the best contrast for detecting the prey rotifers. Experiments used a 25 mm x 25 mm square arena with 3 mm depth. Illumination from above to guide prey capture was provided by a white light (1000 lux) ring of 30 LEDs (designed and fabricated by the Champalimaud Foundation Hardware and Software Platform) positioned around the lens of the camera, 6.5 cm above the arena. Each arena contained a single larva and 50 rotifers (Brachionus plicatilis) and was filmed for 30 minutes. Due to limited availability of breeding fish, prey capture experiments on *sucla2*$^{-/-}$ zebrafish larvae were conducted in a uniform *sirt5*$^{+/-}$ background. Our data did not show any evidence of an effect of this genotype on behavior.

Genotyping was performed after completion of the behavior assays and data processing, allowing for blinding prior to assigning genotypes to the quantified values of each zebrafish. Genotyping Primer pair sequences were used as follows: sucla2: 5′-GGTGGTCGAGGCAAAGGCAC-3′ (forward) and 5′-CATGCATACAATGTAGTAGCAAACACTCACGAG -3′ (reverse).

The number of rotifers in the entire image (948 pixels by 948 pixels) was determined offline using a MATLAB algorithm (Mathworks, USA). A background model was created by calculating the rolling median of each pixel across a window of 40 frames. After scaling images from 0 to 1, the background model was subtracted from each frame, and a binary image was generated using a threshold of 0.03. Connected components in the binary image were identified using the "bwconncomp" function, and the area of each component was taken from the "Area" statistic returned by the "regionprops" function. The histogram of particle areas for each fish exhibited a bimodal distribution, with smaller particles attributed to noise and larger particles corresponding to rotifers or the fish. A size threshold for detecting rotifers was set automatically for each experiment, corresponding to the location of the minimum between the noise and rotifer peaks in the size distribution. Additionally, particles smaller than 5 pixels or exceeding 75 pixels in size were excluded as they were outside the possible range of rotifer sizes and represented either noise, the fish, or floating particles in the medium. Rotifers were counted for 1 frame every 2 seconds. To confirm the tracking accuracy of rotifer counts over time, a control dish with rotifers but no fish was included in the experiment. Frame-to-frame variations in rotifer count occurred due to noise, image overlap with other rotifers, attachment to each other, or occlusion by the arena edges, but the average count in these dishes remained stable throughout the experiment.

mtDNA quantification

Genomic DNA from *sucla2*$^{-/-}$ zebrafish larvae and wild-type control was extracted and precipitated with ethanol. MtDNA content was determined by qPCR as above, using *mt-co1* as the mitochondrial gene target and *polg1* gene as a reference for nuclear DNA content. The following primers were used: polg1-Fwd: GAGAGCGTCTATAAGGAGTAC, polg1-Rev: GAGCTCATCAGAAACAGGACT, mtco1-Fwd: ACTTAGCCAACCAGGAGCAC, mtco1-Rev: TCGGGGAAATGCCATATCGG.

Metabolomics

Sample preparation was performed as follows: tissues were homogenized in 300 µL of -20°C methanol/water (5:3) using 5-mm stainless steel beads in a tissue grinder (Qiagen TissueLyser II) for 1:50 min:sec at 20 Hz. A fully labelled ^{13}C yeast biomass (50 µL) were added to the homogenate as internal standard. Further, 500 µL of methanol/water (5/3, v/v) (-20°C) and 500 µL of chloroform (-20°C) were added; the samples were agitated for 10 minutes at 4°C in a thermo-shaker (Thermomixer C, Eppendorf), followed by 10 minutes of centrifugation. After extraction, the upper polar phase was recovered. The recovered extract was dried overnight in a vacuum centrifuge at 4°C and 5 mbar, and then stored at -80°C, before analysis. Dried samples were reconstituted in 20 µL of 60% (v/v) acetonitrile/water, and the supernatants transferred into glass vials for LC-MS analysis.

Supplementary Table 1 Antibodies and reagents

REAGENT or RESOURCE	SOURCE	IDENTIFIER
**Antibodies and western blotting reagents**		
Pan anti-Succinyllysine antibody (working dilution 1/500)	PTM biolabs	CAT# PTM-401
Sucla2 (working dilution 1/5000)	Abcam	CAT# ab183513
Tubulin (working dilution 1/10000)	Abcam	CAT# ab6046
Hsc-70 (working dilution 1/5000)	Santa Cruz	CAT# sc-7298
Vdac1 (working dilution 1/500)	Abcam	CAT# ab15895
anti-rabbit IgG HRP-labeled (working dilution 1/10000)	Perkin Elmer	CAT# NEF812001EA
anti-mouse IgG HRP-labeled (working dilution 1/10000)	Perkin Elmer	CAT# NEF822001EA
RIPA Buffer	Sigma Merck	CAT# R0278
Halt protease inhibitor	Thermo Fisher Scientific	CAT# 78429
Pierce BCA Protein Assay Kit	Thermo Fisher Scientific	CAT# 23225
NuPAGE 4 to 12%, Bis-Tris, 1.5 mm, 15-well	Thermo Fisher Scientific	CAT# NP0336BOX

Trans-Blot Turbo Midi 0.2 µm Nitrocellulose Transfer Packs	Bio-rad	CAT# 1704159
Pierce™ ECL Western Blotting Substrate	Thermo Fisher Scientific	CAT# 32209
**Chemicals**		
SYBR green master mix	Thermo Fisher Scientific	CAT# 4385614
Alamar blue dye	Thermo Fisher Scientific	CAT# DAL1025
Tricaine Methanesulfonate	Sigma Merck	CAT# E10521
Zebrafish medium	Instant Ocean® sea salt	CAT# SS15-10
Sodium bicarbonate	Sigma Merck	CAT# S5761
DMSO	Sigma Merck	CAT# D8418
FCCP	Sigma Merck	CAT# C2920
Nicotinamide	Sigma Merck	CAT# N0636
Nicotinamide riboside chloride	Chromadex	CAT# ASB-00014315
Nicotinic Acid	Sigma Merck	CAT#N0761-100G
β-Nicotinamide mononucleotide	Sigma Merck	CAT#N3501
Rotenone	Sigma Merck	CAT# R8875
Antimycin A from *Streptomyces sp.*	Sigma Merck	CAT# A8674
Amplex™ UltraRed Reagent	Thermo Fisher Scientific	CAT# A36006
Lactate Oxidase	Sigma Merck	CAT# L9795
LC-MS-grade methanol	Thermo Fisher Scientific	Cat# A456
LC-MS-grade water	Thermo Fisher Scientific	Cat# W6

LC-MS-grade ammonium hydroxide	Thermo Fisher Scientific	Cat# A470
**Resources and commercial assays**		
Gemma 75	Planktovie	CAT# E106502
XF24 Islet Capture FluxPak	Bucher Biotech	CAT# 101174-100
SeQuant® ZIC®-pHILIC 5µm polymer 100 x 2.1 mm	Merck SeQuant	CAT# 150462
SeQuant® ZIC®-pHILIC Guard 20 x 2.1 mm	Merck SeQuant	CAT# 150437
Maxima SYBR Green qPCR Master	Thermo Fisher Scientific	CAT# K0253
NAD/NADH Assay Kit	Enzychrom	CAT#ECNP-100
Urea Colorimetric Assay Kit	Biovision	CAT#K375-100
**Oligonucleotides**		
HRM primer for *sucla2*-/- genotyping	Invitrogen	5′-CTTGGTTATAAAAGCCCAAGTGC-3′ (forward) and 5′-GAGTAAACGATTCTGACTCCTCC-3′ (reverse)
HRM primer for *sirt5-/-* genotyping	Invitrogen	5′-GGGTGGGTAATTGGGAAGTT-3′ (forward) and 5′-GATGGTCCAGTCCTGGTTTG 3′ (reverse)

polg1	Invitrogen	5’-GAGAGCGTCTATAAGGAGTAC-3′ (forward) and 5’-GAGCTCATCAGAAACAGGACT-3’(reverse)
mtco1	Invitrogen	5’-ACTTAGCCAACCAGGAGCAC-3’ (forward) and 5’-TCGGGGAAATGCCATATCGG-3’ (reverse)
**Experimental models: zebrafish lines**		
sucla2-/-	Nestlé Institute of Health Sciences	*sucla2-/-nei010*
Sirt5-/-	Nestlé Institute of Health Sciences	*sirt5-/-nei004*
Tg(ubi:sirt5;cryaa:zsgreen1)	Nestlé Institute of Health Sciences	*Tg(ubi:sirt5;cryaa:zsGreen1)nei005*
All zebrafish lines can be requested for research purposes from the lab of origin		
**Software**		
R (version 3.5.2)	The R Foundation	https://www.r-project.org/
R Studio	The R Foundation	https://www.rstudio.com/
Prism Graphpad Version 8.4.1 (676)	GraphPad	https://praphpad.com/scientific-software/prism/
ZebraLab™	Viewpoint	https://www.viewpoint.fr/
MetaXpress software	MolecularDevices	

Xcalibur 4.2.47	Fisher Scientific	https://www.thermofisher.com/
**Devices**		
Zebrabox	Viewpoint	https://www.viewpoint.fr/
Flex station3	Molecular Devices	
LighCycler96	Roche Life Science	https://diagnostics.roche.com/
TissueLyserII	Qiagen	https://www.qiagen.com/us/
Trans-Blot Turbo Transfer System	Bio-rad	https://www.bio-rad.com/
Seahorse XF24 Extracellular Flux Analyzer	Agilent	https://www.agilent.com/
Thermomixer C	Eppendorf	https://www.eppendorf.com/
Vanquish UHPLC	Fisher Scientific	https://www.thermofisher.com/
ImageXpress Micro XLS microscope	MolecularDevices	
Orbitrap Fusion Lumos mass spectrometer	Fisher Scientific	https://www.thermofisher.com/

Supplementary References

1. Marques JC, Lackner S, Felix R, and Orger MB. Structure of the Zebrafish Locomotor Repertoire Revealed with Unsupervised Behavioral Clustering. *Current biology : CB.* 2018;28(2):181-95 e5.
