# Supplementary figures and images for: NAD+ and Sirt5 restore mitochondrial bioenergetics failure and improve locomotor defects caused by sucla2 mutations
Source: JCI Insight. 2026 Jan 23;11(2):e181812. doi: 10.1172/jci.insight.181812 (PMC12892911; doi:10.1172/jci.insight.181812)

Figure 4A

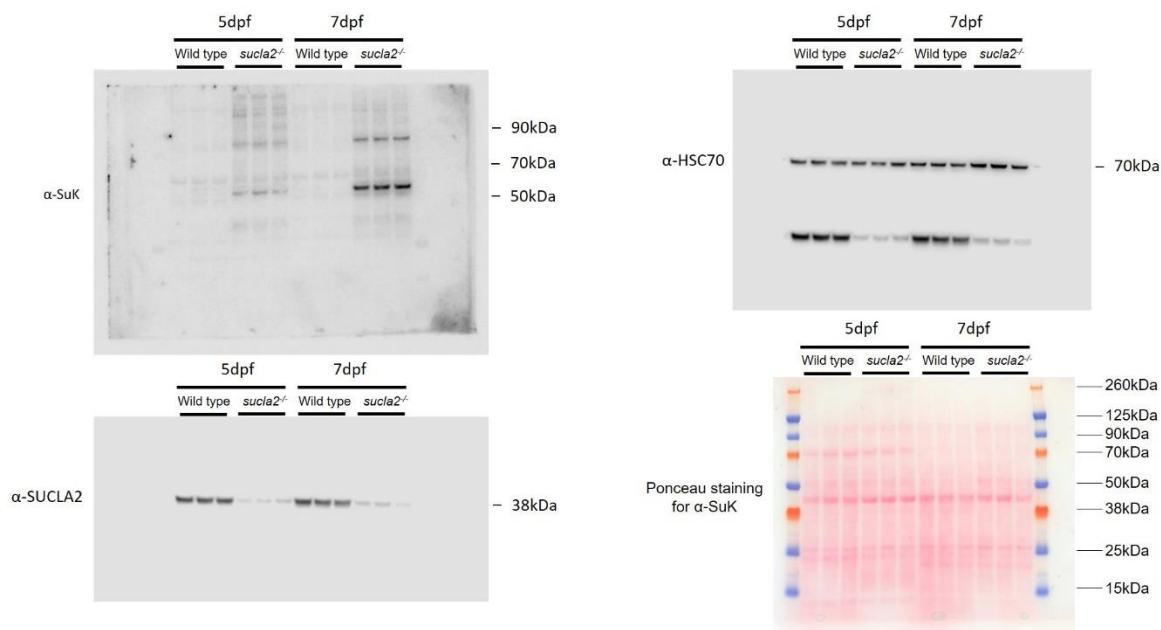

**Supplementary Figure 1C**

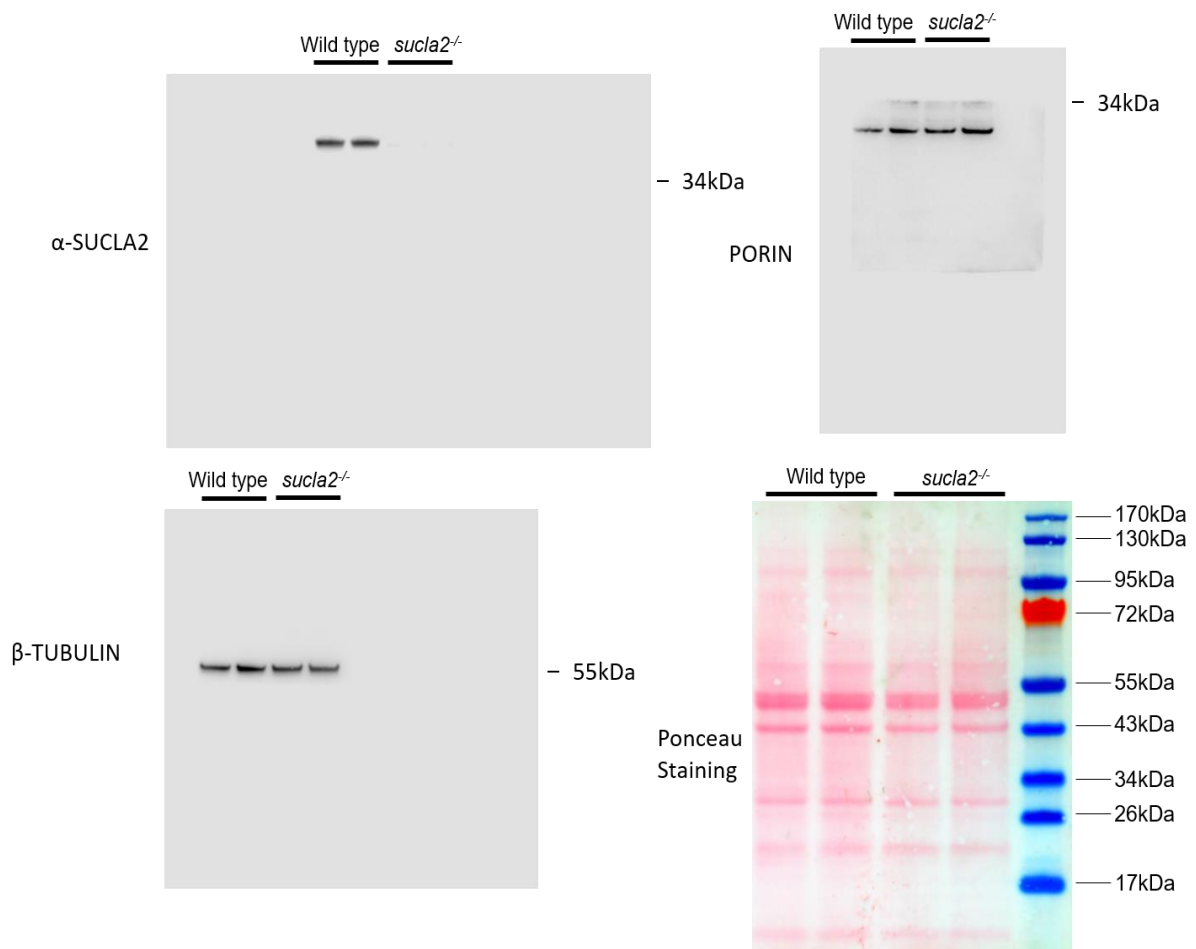

## Supplementary Figure 3B

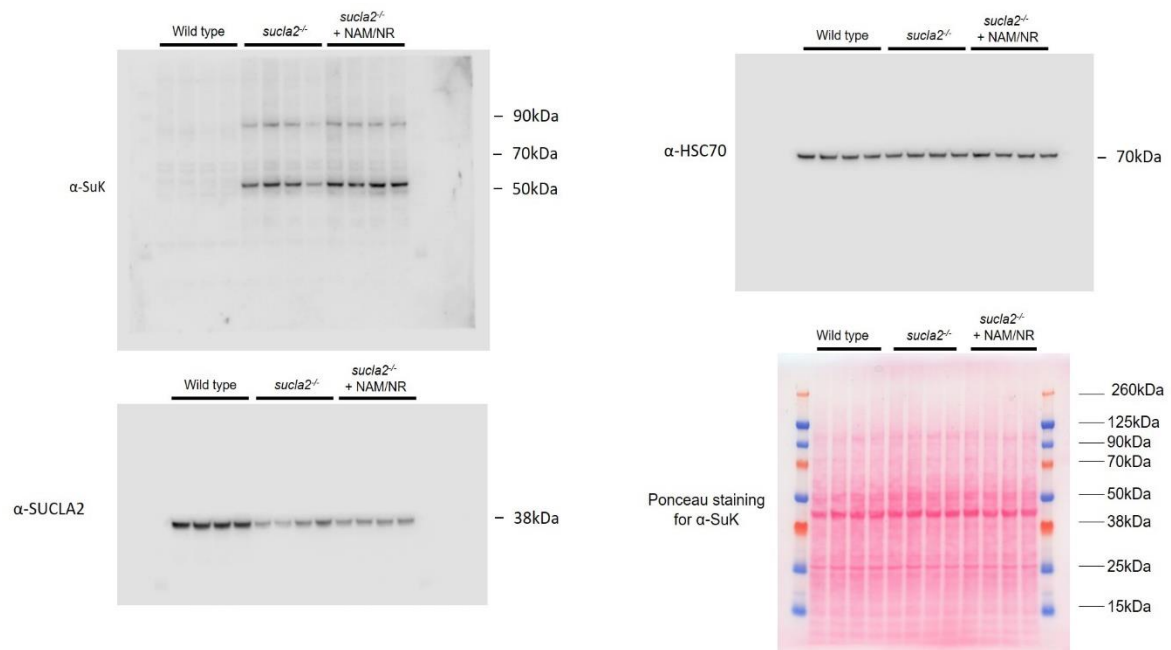

Supplement: Unedited blot and gel images [file jciinsight-11-181812-s236.pdf]
